# PRIMAL: Fast and Accurate Pedigree-based Imputation from Sequence Data in a Founder Population

**DOI:** 10.1371/journal.pcbi.1004139

**Published:** 2015-03-03

**Authors:** Oren E. Livne, Lide Han, Gorka Alkorta-Aranburu, William Wentworth-Sheilds, Mark Abney, Carole Ober, Dan L. Nicolae

**Affiliations:** 1 Department of Human Genetics, The University of Chicago, Chicago, Illinois, United States of America; 2 Departments of Medicine, and Statistics, The University of Chicago, Chicago, Illinois, United States of America; Heinrich Heine University, Germany

## Abstract

Founder populations and large pedigrees offer many well-known advantages for genetic mapping studies, including cost-efficient study designs. Here, we describe PRIMAL (PedigRee IMputation ALgorithm), a fast and accurate pedigree-based phasing and imputation algorithm for founder populations. PRIMAL incorporates both existing and original ideas, such as a novel indexing strategy of Identity-By-Descent (IBD) segments based on clique graphs. We were able to impute the genomes of 1,317 South Dakota Hutterites, who had genome-wide genotypes for ~300,000 common single nucleotide variants (SNVs), from 98 whole genome sequences. Using a combination of pedigree-based and LD-based imputation, we were able to assign 87% of genotypes with >99% accuracy over the full range of allele frequencies. Using the IBD cliques we were also able to infer the parental origin of 83% of alleles, and genotypes of deceased recent ancestors for whom no genotype information was available. This imputed data set will enable us to better study the relative contribution of rare and common variants on human phenotypes, as well as parental origin effect of disease risk alleles in >1,000 individuals at minimal cost.

## Author Summary

The recent availability of whole genome and whole exome sequencing allows genetic studies of human diseases and traits at an unprecedented resolution, although their cost limits the size of the studied sample. To overcome this limitation and design cost-efficient studies, we developed a two step method: sequencing of relatively few members of a well-characterized founder population followed by pedigree-based whole genome imputation of many other individuals with genome-wide genotype data. We show that by sequencing only 98 Hutterites, we can impute 7 million variants in an additional 1,317 Hutterites with >99% accuracy and an average call rate of 87%. Furthermore, parental origin was assigned to 83% of the alleles. Such studies in the Hutterites and other founder populations should yield new insights into the genetic architecture of common diseases, gene expression traits, and clinically relevant biomarkers of disease, and ultimately provide outstanding opportunities for personalized medicine in these well-characterized populations.

## Introduction

Despite decreasing costs of whole exome and whole genome sequencing, the role of rare genetic variants in common disease risk remains hard to assess due to the very large sample sizes required for such studies [1,2]. Therefore, approaches that allow accurate imputation of rare variants to large numbers of individuals based on the sequences of relatively few individuals could address this important question at minimal cost. Founder populations are particularly suitable to this strategy because pedigree relationships are either known or can be inferred from genotypes, facilitating imputation approaches that incorporate identity by descent (IBD) relationships between chromosomal segments and improving imputation accuracy. Moreover, variants that occur at low frequency (<5%) or are rare (<1%) in large outbred populations, may occur at common frequencies (>5%) in founder populations due to the bottleneck at the time of their founding followed by random genetic drift effects in subsequent generations. Similar to mutations for rare monogenic disorders reaching relatively common frequencies in founder populations [3–6], subsets of the rare variants contributing to common complex diseases are also expected to occur at higher frequencies in these populations. This provides a unique opportunity to study the relative roles of rare and common variants on common disease risk in individuals exposed to similar environments, which further minimizes the contribution of non-genetic factors to inter-individual variation in disease risk and facilitates identification of disease-associated alleles.

Methodological approaches to genotype imputation fall into two general categories depending on whether they are based on linkage disequilibrium (LD) or on genetic relationships (i.e., pedigrees) [7]. LD-based imputation methods require a reference panel of genotype training data, usually from unrelated individuals, to infer local haplotype structure, and sharing of haplotype stretches are used for filling in missing genotypes [8–11]. These approaches typically result in high call rates at the expense of lower accuracy, especially for rare alleles [12]. In contrast, pedigree-based imputation approaches are more accurate because they rely on identifying regions of IBD sharing among the study subjects [13,14]. However, call rates are typically lower than from LD-based methods, and pedigree-based imputation can be significantly slower to implement due to complex pedigree structures, which often pose limitations on maximum family sizes and minimum relatedness of individuals [15].

To address the limitations of LD- and pedigree-based imputation methods, we developed PRIMAL (PedigRee IMputation ALgorithm), a fast phasing and imputation algorithm, to assign genotypes at 7 million bi-allelic variants that were discovered in the whole genome sequences of 98 Hutterites to an additional set of 1,317 Hutterites who had genome-wide genotypes for ~300,000 common single nucleotide variants (SNVs). We first phased the SNV genotypes using pedigree-based phasing algorithms [16,17] and determined IBD segments between each pair of haplotypes using a Hidden-Markov Model [18]. We then organized IBD segments into an IBD clique dictionary, a novel data structure for efficient IBD lookup queries that enables fast pedigree-based imputation of the variants identified in the 98 genomes. We demonstrate that the accuracy of the algorithm is above 99% regardless of minor allele frequency, with a call rate of approximately 77%. To improve the call rate, the missing genotypes were imputed using the LD-based IMPUTE2 program [11], with the phased haplotypes of the 98 whole genome sequenced Hutterites as the reference panel. The result is a hybrid method that combines the benefits of pedigree- and LD-based strategies to obtain similar accuracy (> 99%), and higher call rates (87.3%). Moreover, using the IBD clique dictionary implemented in PRIMAL, we can infer the parental origin of 83% of alleles. We are also able to impute whole genome genotypes to recent ancestors with no available DNA. The PRIMAL algorithm and software will facilitate genetic studies of rare variants and parent-of-origin effects in the Hutterites and in other founder populations with similar data.

## Materials and Methods

### Ethics Statement

This study was conducted according to the principles expressed in the Declaration of Helsinki. All participants in the experiment provided written informed consent in approval with the University of Chicago Institutional Review Board.

### Sample Composition

The Hutterites originated in central Europe in the 1500s. After a series of migrations and population bottlenecks, they settled in what is now South Dakota in the 1870s, and currently live on communal farms in the northern U.S. plains states and western Canadian provinces [19]. At present, there are over 14,000 Hutterites living in South Dakota, all of whom are descendants of just 64 founders and related to each other with a mean kinship coefficient of 3.4% [20]. This study includes 1,415 Hutterites who previously participated in one or more of our studies of Mendelian and common diseases and associated phenotypes (e.g., [5,21]). These individuals are related to each other through multiple lines of descent in a 3,671-person minimum pedigree.

### Framework Genome-Wide Genotypes

We genotyped DNA from Hutterite individuals using one of three Affymetrix arrays (500k, 5.0 and 6.0), as previously described [21,22,23]. As part of our quality control (QC) process, we removed SNVs with five or more Mendelian errors, Hardy-Weinberg p-values < 0.001, or call rates <95%, resulting in 332,242 SNVs present on all three platforms. The final sample included 1,415 Hutterites with genotype call rate > 95%. We used the subset of 271,486 SNVs with minor allele frequency (MAF) ≥ 5% for phasing and imputation in this study. These SNVs are referred to as the “framework SNVs”, and genotyped individuals for whom both parents were not genotyped are referred to as the “quasi-founders” of this sub-pedigree.

### Whole Genome Sequencing and QC

Ninety-eight Hutterites were selected from the 1,415 for whole genome sequencing (WGS) to maximize their relatedness to the other 1,317 Hutterites (and thus leading to high pedigree-based imputation call rates), while minimizing the pairwise relatedness among the 98 (to reduce the amount of redundant sequencing). To achieve this we used a greedy algorithm described elsewhere [16] where subjects were selected sequentially to maximize the average kinship to the non-sequenced individuals, while imposing a kinship smaller than 0.1 with the sequenced individuals. Sequencing was performed by Complete Genomics, Inc. (Mountain View, CA). A total of 18.2 million variants (14.0M SNVs, 2.7M insertions, 1.4M deletions; Table 1) were discovered in the 98 WGS, including 11.6 million variants (9.2M SNVs, 1.3M insertions and 1.1M deletions) for which both alleles were called as high quality by Complete Genomics. Using the 332,242 SNVs, the concordance between the genotypes from the whole genome sequences and those determined by genotyping with the Affymetrix arrays was 99.8%.

**Table 1:** Variant summary.

Variant Type			Call Rate Cutoff	Mean Error Rate	Total Variants	Variants Not Passing QC	Variants Passing QC
Non-singletons	RS	SNVs	0.9	0.12%	6,879,488	1,332,939	5,546,549
		Insertions	0.9	0.14%	363,973	259,472	104,501
		Deletions	0.9	0.13%	352,563	222,185	130,378
	Novel	SNVs	0.99	0.68%	1,330,086	667,306	662,780
		Insertions	0.99	0.97%	577,508	547,690	29,818
		Deletions	0.99	0.42%	306,776	232,648	74,128
Singletons	RS	SNVs	0.99	0.42%	277,650	89,448	188,202
		Insertions	0.99	1.37%	28,185	27,089	1,096
		Deletions	0.99	0.50%	24,822	21,613	3,209
	Novel	SNVs	0.99	0.14%	5,522,109	5,302,013	220,096
		Insertions	0.9	1.99%	1,756,253	1,728,591	27,662
		Deletions	0.99	1.50%	756,632	736,385	20,247
Total		SNVs	-	0.27%	14,009,333	7,391,706	6,617,627
		Insertions	-	1.10%	2,725,919	2,562,842	163,077
		Deletions	-	0.51%	1,440,793	1,212,831	227,962
		All Variants	-	0.37%	18,176,045	11,167,379	7,008,666

Variants present in 98 Hutterite genome sequences, shown by singleton (one alternative allele among the 98 sequenced samples) vs. non-singletons, by novelty (variants with and without rs numbers in dbSNP135), and by variant type (SNVs, insertions, deletions). Insertions and deletions are called relative to the NCBI build 37 reference human genome. The mean genotyping calling error rate is estimated using IBD2 segments (see text). QC is based on a combination of using CGI high-quality genotype calls and the shown call rate cutoffs.

To investigate the quality of sequencing-based genotypes for classes of variants (for example, all novel singletons), we developed a pedigree-based method to assess genotyping errors. The method is an extension of the classical Mendelian error checking in families. However, in contrast to Mendelian checks that use parents and their offspring, our approach includes all pairs of related individuals, regardless of the distance of the relationship, using their IBD segments. High confidence IBD2 segments (i.e., IBD = 2, or regions where two individuals inherited the same chromosomal segments from a common ancestor) were previously calculated between each pair of individuals among the 98 Hutterites using the 332,242 framework SNVs [24]. Next, for each sequenced variant, we determined the number of IBD2 segments shared between pairs of individuals that contain the variant and counted the number of discordances (the number of pairs of IBD2 segments in which the genotypes for the variant under investigation did not match). We then estimated the variant calling discordant rate (the proportion of discordances) for each class of variants as the total number of discordances divided by the total number of pairs of IBD2 segments in that category. Discordant rates increased with decreasing call rate, suggesting poorer quality of genotype calls for variants with more missing data. Thus, we determined call rate cut-offs for each variant class to maintain a less than 0.5% discordant rate. This resulted in a final set of variants that included all non-singletons (i.e., variants in which the rare allele occurred at least twice) with rs numbers (in dbSNP135) with call rates > 90% and novel variants (no rs number in dbSNP135) with call rates > 99% (i.e., at most one missing call). Among singletons (variants with one copy of the rare allele in the sequenced subjects), we retained novel insertions with call rates > 90% and all other variant types with call rates > 99% (Table 1, and Fig S2 in S1 Text). The allele frequency distribution and functional annotation of the final set of 7,008,666 variants in the 98 Hutterites with WGS are shown in Fig S2 in S1 Text.

The quality of imputed genotypes was assessed by comparing them to data from a different whole-genome sequencing study in five parent-offspring trios who were among the 1,317 Hutterites in our study [25]. These 15 individuals were sequenced on the Illumina platform at a 10–17x coverage. High quality (as determined by Illumina) genotypes were extracted for all the SNVs imputed using PRIMAL and that passed QC. One of the 15 subjects was sequenced on both platforms and this allowed us to estimate the joint sequencing error rate. Discordance rates between the Illumina sequence-based and PRIMAL-imputed genotypes were calculated as the proportion of differences in genotypes in each of the remaining 14 individuals using these two methods.

### Software

The algorithm described in Results is implemented in software, PRIMAL v1, that is freely available for academic use from the website: https://github.com/orenlivne/ober

## Results

### Pedigree-based Imputation (PRIMAL)

**Fig 1:**
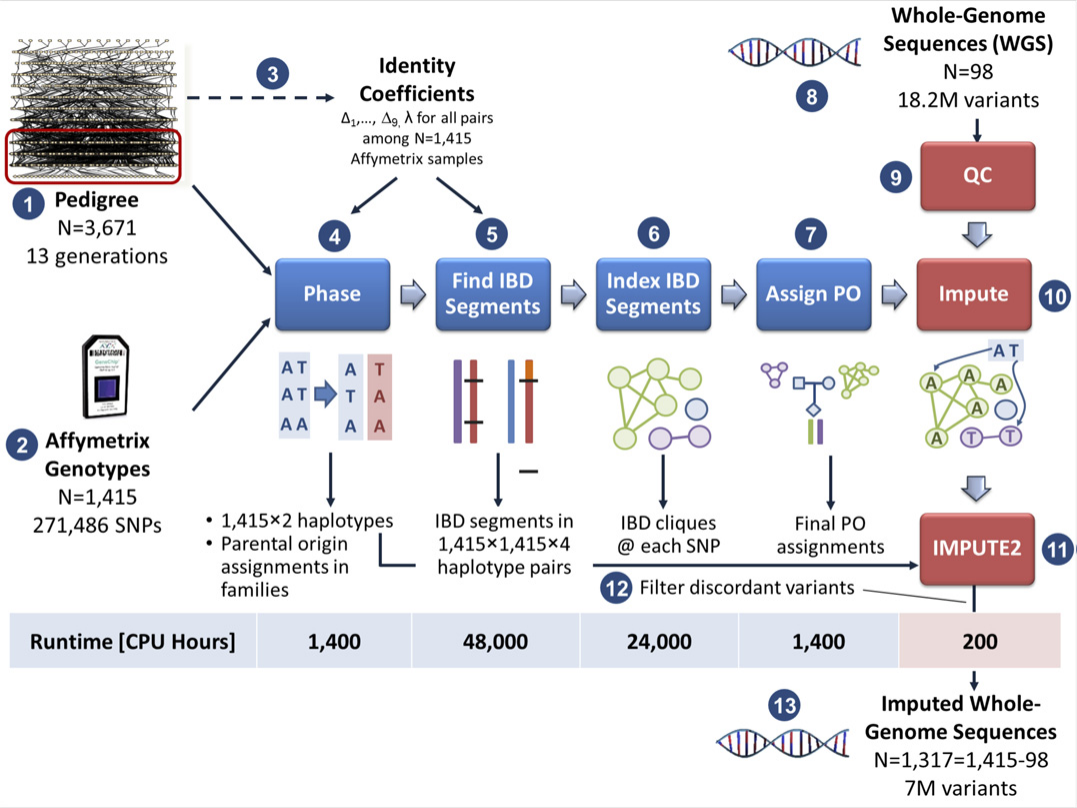
The imputation pipeline. Given a pedigree tree of 3,671 Hutterites (1), 1,415 individuals in the three most recent generations (within the red box) were genotyped with framework markers (2). The first part of the pipeline (steps 2–6) depends only on the framework marker data; the second part (steps 7–9) imputes the whole genome sequence variants. First, estimates of identity coefficients and the transition rate parameter λ [24] between each pair of the 1,415 individuals are calculated (3). The framework genotypes are then phased (4), IBD segments between haplotypes are identified using a HMM (5), and indexed into an efficient data structure consisting of IBD cliques (6). Haplotypes are assigned parental origins consistent across the pedigree using the cliques (7). Then, the whole genome sequences of 98 Hutterites (8) are cleaned using several filters, including a novel generalized Mendelian error check (9), and imputed to the remaining 1,317 Hutterites using IBD cliques (10). Call rates are boosted by imputing as many of remaining genotypes as possible using an LD-based imputation method, IMPUTE2 (11). To ensure that accuracy is not compromised, we calculate the concordance of the shared genotypes between the two methods and keep only variants that are highly concordant (12).

Our imputation algorithm consists of five main stages (Fig. 1, steps 4–8). The first four require only the framework SNVs: (i) phasing; (ii) identifying IBD segments among all haplotype pairs; (iii) indexing IBD segments into a dictionary of IBD cliques; and (iv) assigning parental origin to haplotypes. In the fifth step, we phase the WGS-derived genotypes, and then perform fast pedigree-based imputation of all variants present in the WGS using the IBD clique dictionary.

### Phasing

Our phasing method is similar to the long-range phasing algorithms described by Kong *et al*. [17] and Glodzik *et al*. [13] and to our earlier phasing algorithm for Hutterite genotype data [16], but introduces two key improvements that boost its quality. First, we use a phased proband as a template to phase siblings in nuclear families as in Coop *et al*. [26] (Supplementary Materials S1 Text), and second, we employ a Hidden Markov Model (HMM) similar to the IBDLD model [24] to identify IBD segments between a proband and his/her surrogate parents (Fig S3 in S1 Text). The phasing workflow is outlined in Fig S3 and described in detail in S1 Text. Using this approach, only 0.5% of the framework genotypes remained unphased, 99.2% of the genotypes were correctly phased, and the remaining 0.3% of the framework genotypes were discordant.

### IBD Segment Identification

During the phasing step, IBD segments are identified, but only between the individual being phased and his/her surrogate parents. Therefore, we created a complete IBD dictionary by identifying IBD segments between each pair of the 2×1,415 = 2,830 haplotypes in the sample (S1 Text). Computational complexity prevented us from using available software to estimate IBD segments in related individuals [27–29]. Our HMM is the haplotype analogue of the genotype HMM used for phasing, and is similar to the HBD-HMM developed previously [18]. However, only kinship coefficients are used instead of condensed identity coefficients. The complexity is quadratic in the number of samples, but the hidden constant is small because only two states (IBD or not IBD) are possible instead of the nine in the genotype HMM (Table S1 and S1 Text).

A total of 97,821,947 IBD segments were identified among the 1,415 Hutterites (~1.1 segment per haplotype pair on average, because there are 2830×2829/2 = 4,003,035 individual pairs and 22 chromosomes). To verify the overall quality of the detected IBD segments, our fraction of the genome covered by IBD segments was compared to the fraction calculated by IBDLD [24]. The methods were concordant (correlation coefficient r = 0.96 with a slope of β = 1.01) and the length distribution followed an exponential distribution, in accordance with theory [30].

### IBD Segment Indexing into Cliques

We organize IBD segments in an *IBD segment index* data structure, which consists of a set of IBD *cliques* at each SNV and allows a quick O(1)-time queries of whether a pair of haplotypes is IBD at a certain SNV.

**Fig 2:**
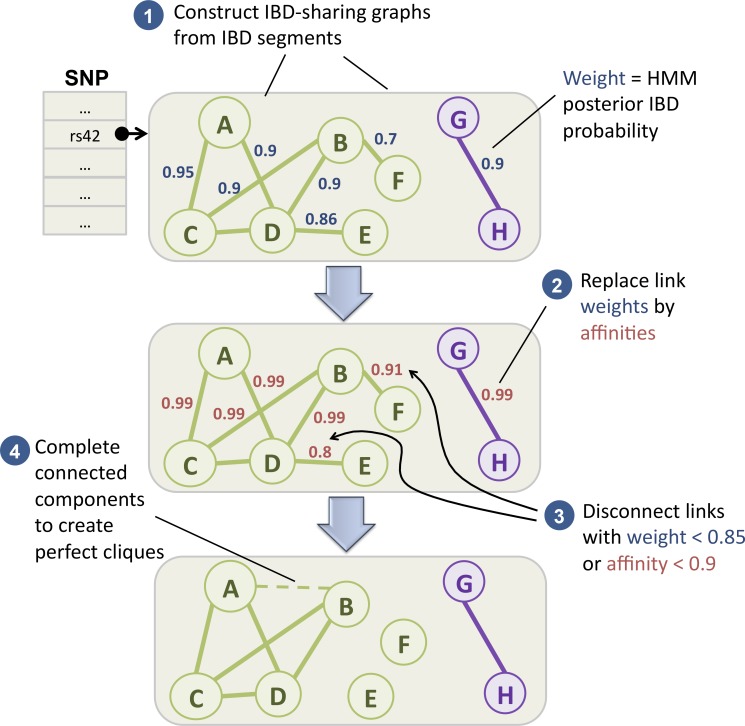
Partitioning an IBD-sharing graph into cliques. (1) IBD segments are indexed into a graph at each SNV. Nodes represent haplotypes (denoted A-H). Each pair of haplotypes that share an IBD segment at the SNV is connected with a link whose weight equals the HMM posterior probability. (2) Link weights are replaced by affinities. Links with small original weight or affinity are removed (3); all nodes within each of the resulting connected components are connected (4).

At each SNV, we build a weighted, undirected pairwise IBD graph G (Fig. 2) whose nodes are the 2,830 haplotypes of the 1,415 Hutterites, an edge indicates the two haplotypes are IBD, and the edge weight is the HMM posterior probability of IBD (S1 Text, Eq. (19c)). Large weights are thus given to haplotype pairs that have a higher probability of being IBD.

Because IBD is a transitive relation, G must be a union of disjoint *cliques* (fully connected sub-graphs), one for each ancestral haplotype present in the population. In practice, G is a perturbation of a clique union due to very low HMM certainty near segment ends and genotyping errors, and we would like to recover a “reasonable'' set of cliques from it. Cluster editing methods (see for example [31]) find the minimum number of edges (or total edge weight) that need to be added or removed to transform G to a clique union. This is an NP-hard problem, and practical heuristic-based algorithms run in superlinear time in the number of edges. We chose a different heuristic inspired by the graph algebraic multigrid literature [32–34] that resulted in good imputation cross-validation accuracy and has linear complexity (S1 Text). First, we calculate new edge weights called affinities that measure the connectedness or affinity between the graph *neighborhoods* of the nodes (Fig. 2). A large affinity means that the nodes share many common neighbors, i.e., they are connected via many short paths. Next, we removed graph edges with weight < 0.85 or affinity < 0.9. These thresholds were chosen to minimize imputation errors in a cross-validation of several framework SNVs representing the entire MAF spectrum. Finally, each of the resulting graph’s connected components is transformed to a clique by adding links between all nodes that are not yet connected (Fig. 2 and Fig S4 in S1 Text). This method worked well for our data set, and these thresholds should be good default values for other data sets. However, threshold determination and a comparison with other clique-generation methods undoubtedly need to be further investigated in a future research.

The use of cliques significantly speeds up imputation because all haplotypes in a clique are imputed simultaneously. In addition, cliques allow the derivation of the maximum call rate obtainable per SNV from imputation, which is the ratio of the number of haplotypes in cliques containing haplotypes of sequenced individuals to the total number of haplotypes. The predicted imputation rate was 85% ± 9% for the framework SNVs. Note that, using pedigree-based imputation, the accuracy approaches 100% (because we rely on Mendelian rules).

### Parental Origin (PO) Assignment

Genotyped individuals are considered “quasi-founders” if either of their parents were not genotyped. Haplotypes of non-quasi-founders can be automatically labeled as paternal and maternal because their parents are included in our sample and haplotypes are assigned using Mendelian rules. However, because the quasi-founders do not have genotyped parents, the parental origin of the quasi-founder haplotypes is assigned in two stages.

First, during phasing, we do not determine which haplotypes are paternal and maternal, but we ensure that the first haplotype of every child comes from the same parent (arbitrarily denoted A), and the second haplotype from the other parent (arbitrarily denoted B). This is achieved using the following steps:

a) Regions of the children’s haplotypes are assigned to four different “bins” (illustrated as four colors in Fig S5 in S1 Text) that represent the four parental haplotypes. Regions that are IBD are in the same bin, under the constraint that the number of recombinations be minimized.
b) There are three possible assignments of four parental haplotypes to parents A and B. For each assignment, we calculate for each child C with haplotypes C_1_, C_2_ a separation measure as follows: let F_1_ be the fraction of C_1_ covered by A’s haplotypes plus the fraction of C_2_ covered by B’s haplotypes, and F_2_ be the fraction of C_1_ covered by B’s haplotypes plus the fraction of C_2_ covered by A’s haplotypes. The separation is the ratio max(F_1_,F_2_), which measures how decisively C’s haplotypes can be identified as paternal or maternal haplotypes.
c) We pick the parental assignment that maximizes the minimum child separation, and order C_1_, C_2_ in all children so that the first always corresponds to parent A and the second to parent B. The separation measure is defined in S1 Text.

**Fig 3:**
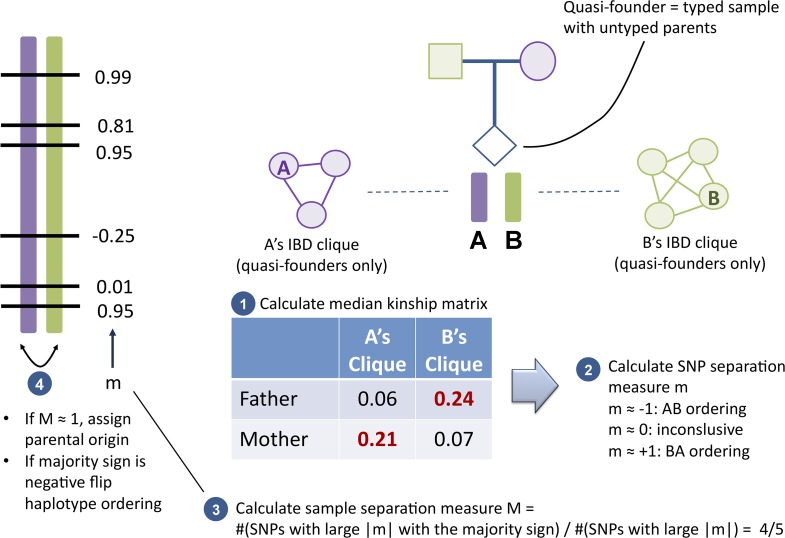
Parental origin assignment process. For a given quasi-founder, we denote his/her haplotypes by A and B, and (by convention) the first is paternal and the second is maternal. At each SNV, we calculate a 2×2 matrix of kinships (Step 1) between each of the proband’s parents and each subject in the A and B IBD cliques. Using these, we generate a parental haplotype separation measure m (Step 2). If m≈1, A and B are already correctly ordered; if m≈-1, they should be swapped. If the majority of the SNVs agree on the same swapping (indicated by a sample separation M sufficiently close to 1 in Step 3), we assign paternal origin and reorder A and B accordingly (Step 4).

Next, after parental origin is assigned to haplotypes within each nuclear family (both parents and their children), we calculate a different separation measure at each SNV for each quasi-founder C. Let 1 and 2 denote the child’s haplotypes, C_1_ and C_2_ the corresponding IBD cliques, and A and B representing C’s untyped parents. For each parent and each clique, we calculate the median of the set of kinship coefficients between the parent and all quasi-founders in the clique that are not siblings of the proband (the quasi-founder in question), resulting in a 2×2 matrix (Fig. 3; siblings and non-quasi-founders are excluded to minimize bias). For each SNV, indexed by s, we define a separation measure m(C, s) (precisely defined in the Supplementary S1 Text, Eq. (4)) such that-1 ≤ m(C, s) ≤ 1. The measure approaches-1 when the off-diagonal matrix elements are much larger than the diagonal elements, and approaches 1 when the diagonal elements dominate. If the proband is properly phased, m(C, s) must be consistently positive or negative across the chromosome. We consider only “informative variants” as those where |m(C, s)| > 0.25 is separated from 0. Suppose there are n_+_ informative variants with m(C, s) > 0 and n_-_ with m(C, s) < 0; the sample separation measure M(C) is defined as max(n_+,_ n_-_) /(n_+_ + n_-_). That is, the fraction of variants exhibiting the “majority sign”. We assign parental origin when M(C) > 0.75. Using this approach we were able to assign parental origin to 76% (313 out of 411) of the quasi-founders’ chromosomes, with 279 having M(C) > 0.99 (Fig S6 in S1 Text). Including non-quasi-founders, we were able to assign parental origin to 93% of the sample.

### Pedigree-based Imputation

Once the IBD clique dictionary is constructed, imputation is performed separately and in parallel for each variant present in one or more of the 98 whole genome sequences. The main idea behind the approach is that each sequencing-based allele that is phased on a particular haplotype can be imputed to all the haplotypes in its IBD clique. First, homozygous genotypes are phased, and the alleles and indices of the two haplotypes are placed into a queue. We remove the first haplotype from the queue, and impute all haplotypes in its IBD clique with the same allele. If these include haplotypes of heterozygous genotypes in the 98 sequenced individuals, they can now be phased. For each such individual, we add its other haplotype index and allele to the end of the queue. The next entry in the queue is then similarly processed, except that, when there is conflicting allele information within a clique (when a two-third majority vote does not exist), no haplotype is imputed. We process queue entries one by one until the queue becomes empty.

Using this approach, we imputed 7M variants (Table 2, columns 4–5) in about 75,000 CPU on Beagle, a 150 teraflops, 18,000-core Cray XE6 supercomputer at the Computation Institute, a joint initiative between The University of Chicago and Argonne National Laboratory [35]. Finding and indexing IBD segments into cliques takes the majority of computing time in the PRIMAL pipeline. The dominant complexity term is O(n^2^s), where n = 1415 is the number of genotyped individuals and s = 271,486 is the number of framework markers (S1 Table in S1 Text, columns 2–3).

**Table 2:** Imputation performance.

Metric	PRIMAL, Cross-Validation	PRIMAL WGS Variants	PRIMAL+LD WGS Variants
# Variants imputed	53,861	7,008,666	7,008,666
# Samples	1,317	1,317	1,317
Concordance	99.8 ±. 01%	-	99.3%*
Het concordance	99.8 ±. 01%	-	99.3%*
Phasing rate	99.6 ±. 01%	99.4%	99.4%
Allele call rate	91.4 ± 8%	91.6%	95.1%
Genotype call rate	75.5 ± 14%	76.2%	87.3%
PO assignment rate	80.0 ± 15%	80.0%	83.0%

PRIMAL and PRIMAL combined with LD-based imputation (PRIMAL+LD) performance and calls rates for the 1,317 Hutterites whose genomes were not sequenced. The concordance and het concordance figures marked by asterisks were based on the concordance of the PRIMAL and LD-based imputation on the set of genotypes called by both. Cross-validation SNVs were chosen from the framework SNVs as explained in the text.

The overall genotype call rate was 76.2%. The mean individual call rate was 75.5%; 547 out of 1317 individuals (41%) had call rate ≥ 80%. Call rates were higher in regions with higher framework SNV density, lower recombination rate and farther from the telomeres (Fig S7a in S1 Text). Fig S8a in S1 Text shows that the MAF distributions of European ultra-rare SNVs (MAF = 0 in the 1000 genomes CEU database) are comparable in both the 98 sequenced Hutterite sample set and the 98 sequenced + 1,317 imputed Hutterites (n = 1,415). Furthermore, we compared the Alternative Allele Frequency (AAF) in the Hutterites and CEU sample set. The Hutterite and CEU AAF were highly correlated (Fig S8b-d in S1 Text). Out of 6,715,275 variants that were not A/T or C/G SNVs, 5,299,330 had similar CEU and Hutterite AAFs (absolute difference < 0.1); there were more variants with larger AAF in the Hutterites than in CEU compared to the opposite case (880,912 *vs*. 534,012 variants).

### Cross Validation

To check the accuracy of PRIMAL imputed genotypes, their concordance with the framework genotypes was assessed. First, we phased the framework (Affymetrix) genotypes, identified IBD segments and indexed them into cliques. We then masked the framework genotypes of the 1,317 individuals whose genomes were not sequenced, imputed the framework genotypes, and calculated the concordance between the imputed and true genotypes over a sample of 53,861 framework SNVs (sorted by base-pair position, every 5^th^ framework SNV was picked instead of using all SNVs to save computing time). The concordance was close to a 100% regardless of MAF (Fig S7c in S1 Text). In addition, we also tested for heterozygote concordance rate within the variants with MAF < 5% because the concordance over all genotypes would be high even if they were randomly imputed. The heterozygous concordance also approached 100%.

### Comparison with Genotypes from an Independent WGS Experiment

We also calculated concordance rates between imputed genotypes based on the 98 Hutterites sequenced by Complete Genomics and genotype calls for 14 Hutterites who were sequenced on the Illumina platform as part of a separate study [25]. The concordance rate for each subject was larger than 99% (the concordance rates ranged from 99.3% to 99.8%) with an overall average of 99.7%. This overall rate is very similar to the rate of concordance obtained from the subject sequenced on both platforms.

### Increasing Call Rates Using LD-based Imputation

The use of cliques significantly speeds up imputation and also allowed us to determine that the maximum predicted imputation rate is 85% for the framework SNVs. However, while genotypes imputed by PRIMAL had high accuracy, the call rate (77%) is lower than the maximum predicted rate, most likely due to imperfect phasing of variants without a consensus allele. To mitigate this problem, we filled in as many genotypes as possible for the remaining 23% of variants using LD-based imputation. We chose IMPUTE2 [11] because of its ease of use, high speed and high imputation accuracy. Importantly, we used the high quality pedigree-based phased haplotypes from the 98 whole genome sequenced individuals as the reference panel. This boosted the IMPUTE2 accuracy (evidenced by the measures described below) and reflects the accuracy of our phasing. To obtain data that are consistent in format and accuracy to those generated by PRIMAL, IMPUTE2 genotype probabilities were converted to hard genotype calls only if the maximum probability among the three possible genotypes was > 99%; otherwise, they were not called. When using this criterion, the concordance rates between IMPUTE2 genotypes and those based on sequencing in the 14 individuals range between 99.5 and 99.8% with an overall average of 99.7% (identical to PRIMAL).

As a QC check on this second round of imputation, we calculated overall as well as heterozygote concordance rates between PRIMAL and IMPUTE2 imputed SNVs. All genotypes called by both methods and called as heterozygous by at least one of them were included. IMPUTE2-imputed genotypes were retained only if the heterozygous concordance rate was ≥ 99% and the MAF ≥ 1% (heterozygous concordance rate drops significantly for variants with MAF <1%—Fig S9 in S1 Text). Finally, the PRIMAL+IMPUTE2 combined method yielded an overall call rate of 87.3% with > 99% estimated accuracy (Table 2).

We also used LD-based imputation to increase the parent of origin (PO) assignment for each allele. First, we created a data set with twice the number of samples (2N). For each subject, we created “paternal haploid” and “maternal haploid” sets. For unphased genotypes, the haploid entries were set to missing. We ran IMPUTE2 on the haploid data set. We then assigned parental origin to each genotype called by IMPUTE2 in the original data set only if both the PO of the paternal and maternal haplotypes were imputed with maximum probability > 99% and were compatible with the genotype. PRIMAL alone assigned PO to 80% of alleles, but with IMPUTE2 directly imputing from PO-assigned haplotypes, we increase the PO call rate to 83%.

## Discussion

Despite trends over the past nearly 20 years toward genetic association studies in large case-control samples [36], there have been strong arguments for, and a recent re-appreciation of the advantages of family studies for understanding the genetic architecture of complex phenotypes [37–39]. For example, family-based studies are particularly well suited for discovery of rare disease-associated variants and revealing parent-of-origin effects while minimizing potential confounding due to population substructure and genetic and environmental heterogeneity. Moreover, the family structure itself allows more extensive quality control checks of genotype data and ultimately more accurate genotype calls. Now, in the era of whole exome and whole genome sequencing, studies in families and founder populations offer a new, powerful framework for mapping studies because the genome or exome sequences of relatively few ‘founders’ are needed to impute highly accurate whole genome genotypes to other members of the pedigree with only framework genotypes.

We describe in this paper a fast phasing and computationally efficient imputation method (PRIMAL) that combines the advantages of pedigree-based and LD-based methods and obtains accurate genotypes (>99%) and high (87%) call rates in 1,317 related Hutterites using whole genome sequencing data on only 98 related individuals, providing unprecedented coverage of genetic variation in a population sample with extensive phenotyping and demographic data. The call rates and, to a lesser degree the concordance rates, are correlated to the degree of relatedness between the imputed individuals and the sequenced subjects. Fig S16 in S1 Text illustrates these relationships, and suggest that the rates are mostly influenced by the few sequenced subjects who are most related to the imputed individual. Note that similar accuracy can be achieved using IMPUTE2 (as detailed above), with a call rate of 84% when restricting to the high quality called genotypes.

In addition, PRIMAL allows accurate parent-of-origin assignments for each allele as well as imputed genotypes of recent ancestors (or other members of the pedigree) with no DNA or available genotype information. This additional information is unique to this approach, and is crucial for many analyses, such as those looking for parent-of-origin effects in associated variants, and imprinting. PRIMAL can be applied to other founder populations or to large families to provide accurate and nearly complete genotype coverage for relatively very small cost and minimal computation time. The quantity and quality of the genotypes generated using PRIMAL will depend on several factors including the family structures, the extent of IBD sharing between the reference and target subjects, and the quality of framework genotypes that are used for inferring the IBD cliques.

In addition to comprehensive surveys of the effects of all variants present in the Hutterite genomes on risk for common and Mendelian diseases and on disease-associated quantitative phenotypes, these data will facilitate association studies with the > 460,000 variants that are rare (<1%) in European populations but have risen to common (>5%) frequencies in the Hutterites and investigations of the effects of maternally-inherited versus paternally-inherited alleles on disease risks and quantitative trait values, and will allow the incorporation of the additional information from IBD sharing in more efficient genetic association studies. Such studies in the Hutterites and other founder populations should yield new insights into the genetic architecture of common diseases, gene expression traits, and clinically relevant biomarkers of disease, and ultimately provide outstanding opportunities for personalized medicine in these well-characterized populations.

## Supporting Information

S1 Text Supplementary methods showing detailed information on phasing and IBD estimation. (PDF)
