# Supplementary material for: PRIMAL: Fast and Accurate Pedigree-based Imputation from Sequence Data in a Founder Population
Source: PLoS Comput Biol. 2015 Mar 3;11(3):e1004139. doi: 10.1371/journal.pcbi.1004139 (PMC4348507; doi:10.1371/journal.pcbi.1004139)
Supplement: S1 Text — (PDF) [file pcbi.1004139.s001.pdf]

PRIMAL: Fast and Accurate Pedigree-based Imputation from Sequence Data in a Founder Population: Supplementary Materials*

O. E. Livne[1], L. Han[1], G. Alkorta-Aranburu[1], W. Wentworth-Sheilds[1], M. Abney[1], C. Ober[1] and D. L. Nicolae[1,2,3]

[1]Department of Human Genetics, The University of Chicago, Chicago, IL
[2]Department of Medicine, The University of Chicago, Chicago, IL
[3]Department of Statistics, The University of Chicago, Chicago, IL

*Address Correspondence to Dan L. Nicolae, nicolae@galton.uchicago.edu

Contents

Supplementary Methods **3**
Phasing . . . 3
Step 3: Parent-Child Pairs in Nuclear Families . . . 3
Step 4: Children Comparison in Nuclear Families . . . 4
Step 5: IBD between Children in Nuclear Families . . . 4
Step 6: IBD between Proband and Surrogate Parents . . . 6
IBD Estimation Approach . . . 6
Single-Locus IBD Estimation . . . 7
IBD Estimation in a Segment . . . 10
IBD Cliques at a SNV . . . 13

Tables **16**

Figures **21**

Supplementary Methods

Phasing

Our phasing method is similar to the long-range phasing algorithm by Kong et al. [9] and to our earlier approach for phasing Hutterite genotype data [15], but introduces several key improvements that boost its quality. In our method, phasing consists of six steps (Figure S3) that incrementally phase more single nucleotide variants (SNVs) and including a larger sample, starting with individuals then moving to parent-offspring trios to nuclear families and eventually to the entire pedigree. The later steps are more computationally costly.

In the first step we apply Mendelian logic to phase all SNVs for which the proband (Figure S3, Step 1) or one of his/her parents is homozygous (Step 2).

Step 3: Parent-Child Pairs in Nuclear Families

Next, we consider parent-child pairs, in whom $> 50\%$ of the parent's heterozygous SNVs are already phased so that his/her two haplotypes can be delineated (Figure S3, Step 3). The corresponding child's chromosome is a recombinant of these haplotypes. We search for long ($\geq$ 400 SNVs) identity-by-state (IBS) segments between each parental haplotype and the child's haplotype, allowing for localized differences due to potential genotyping errors. First, in each window of 5 consecutive SNVs, if there exists only one non-IBS SNV, it is ignored. Long IBS segments are then declared IBD, and used to phase the parent and child. This allows us to phase triply-heterozygous SNVs that were phased in a parent (because the parent is itself a child in another trio). Our segments are shorter than in previous works [9, 6] (400 SNVs vs. 1000 SNVs) because we only rely on IBS to identify IBD segments in first-degree relatives, where the mean IBD segment length is longer ($25cM$ [4]); for more distant relatives, we employ a more precise Hidden Markov Model (HMM) strategy, cf. Sec. IBD Estimation. Moreover, IBS segments may not cover some short regions around recombination sites because not all SNVs are informative (either the parent is homozygous, or the child or parent has not been phased yet).

After all parent-child pairs are processed, we apply the same procedure to nuclear families to identify all IBD haplotype segments between parents and their children. At each SNV, we thereby determine which family members are IBD, then phase the unphased members using a majority vote of the phased members (Figure S4), as follows. Given a parent P (the father in this example) who is phased at most heterozygous SNVs, call his partially-known haplotypes P_1 and P_2. Let $C_1, \ldots, C_4$ be the partially known paternal haplotypes of his four children. We first determine IBD segments between P and each child using long IBS stretches (Figure S4, step 1), from which we can identify child recombination sites at segment ends (denoted by vertical dashed blue lines; step 2). In the interval between each two sites, we determine the two sets of children that are IBD with P_1 and P_2, respectively (step 3). By transitivity, those children are also IBD among

themselves (dashed lines). For each SNV in the interval and each IBD set, if n_1 phased members of the set have one allele (say, the A-allele in P_1, C_1) and n_2 have the other (say, the T-allele in C_3) with $n_2 < n_1$, then the n_2 alleles are flagged as errors and corrected to allele A (for instance, the T-allele in C_3 is corrected to A), and the majority allele is copied to unphased set members (C_4). If $n_2 = n_1$, all members of the set are zeroed out and flagged as errors, since there is no majority vote. These sets are special cases of IBD cliques described in the main text section "IBD Segment Indexing into Cliques": in both cases we build an IBD-sharing graph between several haplotypes; by transitivity, this graph must be a clique.

Step 4: Children Comparison in Nuclear Families

If a parent is not phased in $> 50\%$ of his/her heterozygous sites, but has $K \geq 3$ children who are $> 90\%$ phased at all sites, we instead use the most-phased child haplotype as a template [6], and examine the IBS state between the template haplotype and the haplotypes of the other $K-1$ children (Figure S3, Step 4). If a single child changes IBS state with respect to the template at a SNV, we infer that a recombination occurred in that child; if $K-1$ children change state at a SNV, it is likely a recombination in the template child (because otherwise $K-1$ children would have a recombination at the same SNV, an event of negligible probability). Knowledge of the template child's haplotype and recombination sites allows us to reconstruct the two parental haplotypes, as well as phase more SNVs in the children as in Step 3. Note that the parental origin of the parent's haplotypes cannot be deduced from their children.

Step 5: IBD between Children in Nuclear Families

In families in which both parents are not genotyped, some of the (quasi-founder) children might now be phased using information on their siblings, but we do not know which of their haplotypes are paternal and which are maternal in origin. To accomplish this, we first classify the haplotypes of phased children into two groups (those inherited from one parent, and those inherited from the other, although we do not yet know their parental origin):

1. We identify IBD segments between each two haplotypes A and B (belonging to different children or to the same child) using a standard HMM described in detail in Sec. IBD Estimation in a Segment, Haplotype vs. Haplotype. Rather than explicitly modeling LD as in IBDLD [7], our strategy is to prune the framework SNVs used in the HMM for LD using a greedy strategy that maximizes the number of SNVs while keeping their remaining pairwise $r^2 < 0.3$ (we pick the first available SNV, remove all SNVs that are in LD with it, then add the closest SNV to the frame, and repeat). The remaining SNVs are treated as probabilistically independent, i.e., the observed genotype at SNV i is assumed to be independent of the IBD states at SNVs $1, \ldots, i-1$. The input to the HMM consists of

- The genotypes of A and B at all framework SNVs.
- The allele frequencies at each SNV (estimated from the genotypes).
- Estimates $\Delta_1, \ldots, \Delta_9$ of the prior probabilities for each condensed identity state that depend only on the known pedigree (Δ_1 is the probability that all four alleles of A and B at a SNV are IBD; etc. [7, Fig. 1]), and an estimate of the transition rate parameter λ, all of which have been previously computed for all the Hutterites individuals in this sample [7].

The output of the HMM is the Viterbi path with the values of the hidden state IBD $\in \{0, 1\}$, indicating whether the haplotypes are IBD or not.

2. Let $r_0, \ldots, r_n$ be the set of recombination points, that is, the set of all segment endpoints, sorted by location, and let m the number of phased children (the chromosome endpoints are included as r_0, r_n). Order haplotypes so that $2i - 1$ and $2i$ correspond to child i, $i = 1, \ldots, n$.

3. We "color" haplotypes with at most 4 colors representing the four parental haplotypes $1, \ldots, 4$. That is, we determine an $(2m) \times m$ matrix P with integer entries $0 \leq p_{i,j} \leq 4$ (0 denotes an unknown region not covered by any paternal haplotype):

   - Start with the initial guess $p_{i,j} = j$; we identify cliques in each P-column (sets C of rows i that are IBD in the chromosomal region $[r_j, r_{j+1}]$) as in Step 3, and within each clique C replace $p_{i,j}$ by $\min_{i \in C} p_{i,j}$. This reduces the number of colors.
   - Recode the colors so that 1 is the most abundant, 2 is the second most abundant, and so on.
   - Recode all colors $k > 4$ to $k = 0$.
   - For each $j = 1, \ldots, n$, swap color 0 with a color $k \leq 4$ in the jth P-column (i.e., update all zero entries $p_{i,j}$ to be k) that minimizes

   $$D(k) := \sum_{j=1}^{n} |P_{i,j} - P_{i,j-1}| + \sum_{j=1}^{n} |P_{i,j+1} - P_{i,j}| \qquad (1)$$

   (for $j = 1$, only the second sum is included; for $j = m$, only the first sum), if there exists a k for which $D(k) < D(0)$.
   - Recalculate cliques in each P-column and swap color 0 with a color $k > 0$ whenever both belong to the same clique. If multiple k's exist, choose the one that minimizes $D(k)$.

4. There are three possible assignments of the 4 paternal haplotypes to two parents A and B. Let A_i be the fraction of genetic distance of haplotype i covered by A's

paternal haplotypes; similarly B_i, for $i = 1, \ldots, 2n$. Let

$$\begin{aligned} R_i &:= (A_{2i-1} + B_{2i})/(A_{2i} + B_{2i-1}), & (2) \\ S_i &:= R_i \text{ if } R_i > 1, \text{ else } 1/R_i, & (3) \\ M(i) &:= f(S_i), & (4) \\ f(x) &:= \operatorname{sgn}(\log(x)) \frac{|\log(x)|}{|\log(x)| + 1}. & (5) \end{aligned}$$

We call M the child's *separation measure.* M ranges between -1 and 1; when M is far from 0, one parental haplotype (color) covers most of a child's haplotype while the other haplotype is mostly covered by a haplotype of the other parent. We choose the assignment that maximizes the minimum separation $M := \min_i |M(i)|$. If $M > 0.25$, we can assign the parental origin of all child haplotype to either A or B based on $M(i)$'s signs. The parental origin provides useful information in itself, but also allows us to phase the unphased children next.

If parental origin assignment is successful ($M > 0.25$), we use an analgous HMM based on genotypes (see Sec. IBD Estimation in a Segment, Genotype vs. Genotype) to identify IBD segments between an unphased child and a phased child. The output of the HMM is the Viterbi path with the values of the hidden state IBD $\in \{0, 1, 2\}$, indicating whether A and B share 0, 1 or 2 alleles IBD. Segments of length ≥ 1 cM in the Viterbi path (Sec. Single-Locus IBD Estimation, Haplotype vs. Haplotype) with hidden state IBD $= 1$ are identified; in each segment, we determine which of the two haplotypes of B fits with A's genotype. When different haplotypes fit different sub-segments of an IBD segment, sub-segments of length ≥ 0.4 cM are output as the final IBD segments and used to phase the unphased child. Haplotype classification into the two parental groups is essential to patching sub-segments into the correct long-range phasing result.

Step 6: IBD between Proband and Surrogate Parents

Finally, for each individual that are $< 95\%$ phased, we search the pedigree for phased surrogate parents [9, 15], first within close relatives and gradually considering more distant relatives up to seven meioses away, invoke the HMM to identify IBD segments, and subsequently phase the proband (Figure S3, Step 6).

IBD Estimation Approach

Our goal is an Identity-by-Descent (IBD) estimation approach that strikes a balance between accuracy and complexity. On one extreme, the paper [15] estimates the IBD of a segment assuming that the IBD state at a SNV is independent of all other SNVs. This single-SNV model is simple and fast, but very crude. On the other extreme lie Hidden Markov Models (HMMs) such as IBDLD [7], which allows the IBD state to change from SNV to SNV and models LD more explicitly.

The key points of our approach are:

- We distinguish between two IBD ascertainment scenarios: genotype vs. genotype (GG) (used during phasing and analogous to [1]), and haplotype vs. haplotype (HH) (used to obtain the full IBD dictionary after phasing). In each case, our goal is to calculate $P(\text{IBD}|\text{data})$.
- We first remove SNVs with missing data in either of the compared subjects.
- Estimation is performed in a *frame*, a subset of pruned SNVs whose pairwise LD $r^2 < 0.3$. Frame genotypes are assumed to be independent. (In practice, the frame with the least amount of missing data is used; IBD segments obtained from different frames can be compared for validation and robustness analysis, but frames cannot be directly combined, as that would violate the independence assumption.)
- We first derive single-locus posterior IBD probability. In the HH case, we improve upon Uricchio et al. [15] by taking into account which allele is shared (Sec. Single-Locus IBD Estimation, Haplotype vs. Haplotype). The GG case is identical to Abney's calculation [1].
- To determine whether a prospective segment is IBD, we need the to define the prior IBD probability for every possible combination of IBD states at the frame SNVs. Uricchio et al. assumed a constant state over the frame, which yields a simple formula, yet this is not a valid assumption in either the HH or the GG case. We also model genotype errors here. Standard HMM tools [12] are applied to calculate the posterior IBD at each marker given the segment data in linear time. The final segments comprise of SNVs whose hidden IBD state is the same.

Our IBD estimation methods are based on Bayes' theorem [8]. In this section, the variable IBD refers to the number of alleles shared between the two individuals compared and can be 0, 1 or 2.

Single-Locus IBD Estimation

Consider a single biallelic SNV with alleles $0, 1$ whose frequencies are p, q, respectively (frequencies are typically estimated from the sample; however, we neglect finite-sample effects here). We write $a \equiv b$ if the alleles a and b are IBD, and $a = b$ if they are IBS.

.1 Haplotype vs. Haplotype

For a single locus, the term "haplotype" is synonymous with "allele". Let $H^{\mathcal{A}}, H^{\mathcal{B}}$ be two alleles randomly drawn from subjects $\mathcal{A}, \mathcal{B}$, respectively (or randomly drawn from the same subject with replacement). Suppose the observed haplotypes are IBS, namely, $H^{\mathcal{A}} = H^{\mathcal{B}}$, and we'd like to know if they are also IBD (if they are not IBS, they are certainly not IBD). Without loss of generality, assume $H^{\mathcal{A}} = H^{\mathcal{B}} = 0$. Let f be the kinship coefficient of $\mathcal{A}, \mathcal{B}$.

We apply Bayes' theorem to the hypothesis event $S = (H^{\mathcal{A}} \equiv H^{\mathcal{B}})$ and evidence event $O = (H^{\mathcal{A}} = H^{\mathcal{B}} = 0)$. The Bayes terms are

$$P(O|S) = P(H^{\mathcal{A}} = 0) = p\,, \qquad P(O|\neg S) = P(H^{\mathcal{A}} = 0, H^{\mathcal{B}} = 0) = p^2\,,$$
$$P(S) = f \text{ (by definition)}\,, \qquad P(\neg S) = 1 - P(S) = 1 - f\,,$$

By Bayes' Theorem,

$$P\left(S|H^{\mathcal{A}} = H^{\mathcal{B}} = 0\right) = \frac{pf}{pf + p^2(1-f)} = \frac{1}{1 + p/\mathrm{OR}}\,, \tag{6}$$

where $\mathrm{OR} := f/(1-f)$ is the *kinship odds-ratio*: the odds of $H^{\mathcal{A}}, H^{\mathcal{B}}$ being apriori IBD. OR acts like a critical allele frequency below which the IBD probability rapidly increases, reflecting the fact that IBS for a rare allele more likely owes to IBD than to random chance.

Uricchio et al.'s IBD estimation [15, Eq. (1)] did not take into account the actual value of allele shared by the haplotypes, i.e., it conditioned on $H^{\mathcal{A}} = H^{\mathcal{B}}$ instead of on $H^{\mathcal{A}} = H^{\mathcal{B}} = 0$. Here

$$P\left(S|H^{\mathcal{A}} = H^{\mathcal{B}}\right) = \frac{f}{f + (p^2 + q^2)(1-f)} = \frac{1}{1 + (p^2 + (1-p)^2)/\mathrm{OR}}\,. \tag{7}$$

(6) and (7) are identical (a) when the two alleles are equally prevalent ($p = 0.5$) – by symmetry; (b) when this is the only allele ($p = 1$), in which case IBS provides no additional information, and $P(S|H^{\mathcal{A}} = H^{\mathcal{B}}) = P(S) = f$; or (c) when $f \to 1$, i.e., the two haplotypes are always identical regardless of the allele.

On the other hand, for $p < 0.5$, (6) becomes much larger as $f \to 0$, as a rare allele occurring twice in otherwise-unrelated subjects is a strong evidence for IBD. Finally, (6) is smaller than (7) when 1 is the major allele, since IBS is less informative when both alleles tend to be equal in all subjects (Figure S11).

.2 Genotype vs. Genotype

Let G^l be the observed genotype of person l, $l = \mathcal{A}, \mathcal{B}$, coded as 0, 1 or 2 (corresponding to the unordered allele pairs 0/0, 0/1 and 1/1), and $G := (G^{\mathcal{A}}, G^{\mathcal{B}})$.

We define three IBD states: 0,1 and 2. Of the nine possible states $S_1, \ldots, S_9$ [10, Table 5.3], $1, 7$ are sub-cases of IBD = 2, $3, 5, 8$ are sub-cases of IBD = 1, and $2, 4, 6, 9$ are sub-cases of IBD = 0. In Table S2, second column, the top two dots represent $\mathcal{A}$'s alleles and the bottom two represent $\mathcal{B}$'s alleles, where the maternal and paternal origins of alleles are ignored. Dots are linked if the corresponding alleles are IBD. For instance, $S = 3$ indicates that both of $\mathcal{A}$'s alleles are IBD, one of $\mathcal{B}$'s alleles is IBD with them, while the other is not. The prior probability of each condensed identity state is given by the vector $\Delta := (\Delta_1, \ldots, \Delta_9)^T$ of identity coefficients $\Delta_i := P(S_i)$, $i = 1, \ldots, 9$, which

is assumed to be known, where T denotes the transpose operator. Note that while the kinship coefficient can be inferred from identity coefficients [10, p. 85],

$$f = \Delta_1 + \frac{1}{2}(\Delta_3 + \Delta_5 + \Delta_7) + \frac{1}{4}\Delta_8\,, \tag{8}$$

the individual Δ_i's cannot be derived from f.

Similarly, let IBS $= 0, 1, 2$ be the number of alleles shared by $G^{\mathcal{A}}, G^{\mathcal{B}}$. Of the nine possible observed genotype states for G, seven indicate IBS ≥ 1 and two indicate IBS $= 0$ (Table S2). Our objective is to compute $P(\text{IBD} \geq 1|G)$, expanded to [1, p. 1582],

$$\begin{aligned} P(\text{IBD} \geq 1|G = \mathbf{j}) &= \sum_{i\in\{1,3,5,7,8\}} P(S = i|G = \mathbf{j}) = \sum_{i\in\{1,3,5,7,8\}} \frac{P(G = \mathbf{j}|S = i)\Delta_i}{\sum_{i'=1}^{9} P(G = \mathbf{j}|S = i')\Delta_{i'}} \\ &= \frac{\sum_{i\in\{1,3,5,7,8\}} b_{i,\mathbf{j}}\Delta_i}{\sum_{i=1}^{9} b_{i,\mathbf{j}}\Delta_i}\,, \end{aligned} \tag{9}$$

for each genotype pair $\mathbf{j} := (j^{\mathcal{A}}, j^{\mathcal{B}})$, $j^{\mathcal{A}}, j^{\mathcal{B}} \in \{0, 1, 2\}$, where $b_{i,\mathbf{j}} := P(G = \mathbf{j}|S = i)$ depend on the allele frequencies and are given in Table S2. This a special case of [1, Table 1] with no missing data. For instance,

$$b_{3,(0,1)} = P\left(G = (0, 1)|S = 3\right) = P\left(G^{\mathcal{A}} = 0, G^{\mathcal{B}} = 1|S = 3\right) = pq\,,$$

because we can draw a single allele from $\mathcal{A}$'s alleles, which determines $\mathcal{A}$'s other allele and one of $\mathcal{B}$'s. This allele equals 0 probability p. The other $\mathcal{B}$-allele is 1 with probability q.

Substituting Table S2 into (9),

$$P\left(\text{IBD} \geq 1|G = \mathbf{j}\right) = \begin{cases} 0\,, & j \in \{(0, 2), (2, 0)\}\,, \\ \frac{\xi_j(p)}{\xi_t(p)+\eta_j(p)}\,, & j^{\mathcal{A}} \neq 2 \text{ and } j^{\mathcal{B}} \neq 2\,, \\ \frac{\xi_{M(j)}(1-p)}{\xi_{M(j)}(1-p)+\eta_{M(j)}(1-p)}\,, & \text{otherwise}\,, \end{cases} \tag{10}$$

with $M(\mathbf{j}) := (2 - j^{\mathcal{A}}, 2 - j^{\mathcal{B}})$ (see Table S3).

.3 Evidence Comparison

Since the posterior probabilities $P(\text{IBD} \geq 1|\text{IBS} \geq 1)$ depend on different identity coefficients in Sections .1 and .2, we assume a kinship model $\Delta = \Delta(f)$ that satisfies [10, p. 85]

$$f = \Delta_1(f) + \frac{1}{2}(\Delta_3(f) + \Delta_5(f) + \Delta_7(f)) + \frac{1}{4}\Delta_8(f)\,, \tag{11}$$

$$\sum_{i=1}^{9} \Delta_i(f) = 1\,, \tag{12}$$

so that they can be compared as functions of p and f. We consider two cases:

- Outbred subjects:

$$\Delta = (0, 0, 0, 0, 0, 0, 0, 4f, 1 - 4f).$$

- Inbred subjects:

$$\Delta = (0.04, 0.04, 0.02, 0.06, 0.04, 0.04, 0.04, 4f - 0.14, 0.86 - 4f).$$

The genotypes corresponding the HH case with shared 0-allele are $\mathbf{j} = (0, 0), (0, 1), (1, 0)$, and $(1, 1)$. Figure S12 illustrates that IBS between two genotype subjects (GG) is usually a stronger evidence for IBD than a pair of haplotypes (HH). This happens because we have assumed that the haplotypes are *randomly drawn* from the subjects, which provides no extra information over genotypes; furthermore, we have implicitly assumed that we do not have genotypes available. Had we used the genotypes, HH and GG would have been identical. In particular, $P(\text{IBD}|G = (1, 1)) \to 1$ as $p \to 1$ (two hets with one rare allele each are always IBD for the rare allele) while in the HH case the posterior probability tends to f. On the other hand, all posterior probabilities are always equal for $p = 0, 1$, because IBS for a very rare allele always implies IBD, while IBS for a very common allele has no effect on our IBD belief.

The HH evidence would have been stronger than GG had we also assumed the knowledge of which parent each haplotype was inherited from, and used the corresponding detailed identity coefficient [10, Fig. 5.2] to express $P(\text{IBD})$. Unfortunately, the uncondensed coefficients are not yet available for the Hutterites data set.

IBD Estimation in a Segment

While a single IBS SNV generally provides some evidence of IBD, as evidenced by Figure S12, a sufficiently long, nearly uninterrupted segment of IBS SNVs makes a much stronger evidence. Starting from the set of all SNVs in the chromosome, we remove SNVs in which either subject has a missing genotype, and further restrict ourselves to a frame of nearly-independent SNVs.

Let n be the the frame size; number the SNVs in the frames from 1 to n with genetic positions $x_1, \dots, x_n$. Let $p_{k,l}$ be the frequency of allele l at SNV k, for $l = 1, 2$.

.1 Haplotype vs. Haplotype

We consider two haplotypes randomly drawn from $\mathcal{A}$ and $\mathcal{B}$. Let $\mathbf{O} := (O_1, \dots, O_n)$ be the vector of observed haplotypes, $\mathbf{H} := (H_1, \dots, H_n)$ the vector of true haplotypes, and $\mathbf{S} := (S_1, \dots, S_n)$ the (hidden) IBD state vector. $O_k = (O_k^{\mathcal{A}}, O_k^{\mathcal{B}})$, where O_k^l is the allele of person l at SNV k; similarly $H_k = (H_k^{\mathcal{A}}, H_k^{\mathcal{B}})$. $S_k = 1$ means IBD, i.e., $H_k^{\mathcal{A}} \equiv H_k^{\mathcal{B}}$, and $S_k = 0$ means "not-IBD". Our goal is to calculate the posterior IBD probability at each marker, $P(S_k = 1|\mathbf{O})$.

We assume that frame SNVs are independent; thus

$$P(\mathbf{O}|\mathbf{S} = \mathbf{s}) = \prod_{k=1}^{n} P(O_k|S_k = s_k)\,. \tag{13}$$

We approximate the IBD state vector along the genome as Markov [2, p. 924]. Thus

$$P(\mathbf{S}=\mathbf{s}) = P(S_1 = s_1) \prod_{k=1}^{n-1} P(S_{k+1} = s_{k+1} | S_k = s_k) \ . \tag{14}$$

To define an HMM, we define the transition probabilities $a_{i,j}^k := P(S_{k+1} = j | S_k = i)$. In analogy to [2, 7], the transition matrix is an infinitesimal rate matrix model $\mathbf{A}^k = \mathbf{A}(x^{k+1} - x^k)$, where

$$\mathbf{A}(x) = (a_{i,j}(x))_{i,j=0,1} = \mathbf{f}\mathbf{1}^T + e^{-\overline{\lambda}x}\left(\mathbf{I}_2 - \mathbf{f}\mathbf{1}^T\right) \ , \tag{15}$$

$\mathbf{I}_n$ is the $n \times n$ identity matrix, $\mathbf{1} = (1,1)^T$, $\mathbf{f} := (1-f, f)^T$ is the unconditional probability of IBD states, f is the kinship coefficient of $\mathcal{A}, \mathcal{B}$, and $\overline{\lambda}$ is the transition rate in either $H^{\mathcal{A}}$ or $H^{\mathcal{B}}$ (for simplicity, we assume this rate is constant over the frame; the method is easily extensible to a local rate, if such is available). (15) satisfies the boundary conditions at $\mathbf{A}(0) = \mathbf{I}_2, \mathbf{A}(\infty) = \mathbf{f}\mathbf{1}^T$.

$\overline{\lambda} = \overline{\lambda}(\mathcal{A}, \mathcal{B})$ could be chosen as the best fit in gene dropping simulations [2, p. 925]. Here we choose a simpler alternative: the transition rate $\lambda(\mathcal{A}, \mathcal{B})$ in a pair of subjects was available to us for all pairs of Hutterites [7]. In particular, if $\mathcal{A}$ and $\mathcal{B}$ have children, the genotype of each child $\mathcal{C}$ comprises of two randomly drawn haplotypes (generally, two recombinant haplotypes) from the four parent haplotypes. Thus $\overline{\lambda}$ can be approximated by the mean $\lambda(\mathcal{C}, \mathcal{C})$ over all children. Since all children share the same inbreeding coefficient f, we further approximate $\overline{\lambda}$ to be a function of f only (Figure S13b). Considering all subjects, we calculate the mean λ over bins of similar f values, obtaining a lookup table for $\overline{\lambda}(f)$ (Figure S13a, red line), and linearly interpolating to any f-values in between. This table only includes small f values, since spouses are typically at least four meioses apart, but the standard deviation in $\overline{\lambda}$ decreases as f increases, and the mean curve is smooth enough to allow a reasonable extrapolation to larger f's. Note that $\overline{\lambda}$ is also a decreasing function of f, as distantly-related spouses result in shorter IBD segments., i.e., a higher reomcbination rate.

Order the possible observed haplotypes as the vector $\mathcal{O} := ((0,0),(0,1),(1,0),(1,1))$ and hidden states by $i = 0, 1$. Let $b_{i,\mathbf{j}}^{(k)} := P(O_k = \mathbf{j} | S_k = i)$. We assume a two-step Markov process $S_k \to H_k \to O_k$. Therefore, the emission probability matrix is the product

$$\mathbf{B}^{(k)} := \left\{ b_{i,\mathbf{j}}^{(k)} \right\}_{i,\mathbf{j}} = \mathbf{B}(p_{k,0})\mathbf{E}(\varepsilon) \, , \tag{16}$$

where

$$\begin{aligned} \mathbf{B}(p_{k,0}) &:= \{P(H_k = \mathbf{j} | S_k = i)\}_{i=0,1,\mathbf{j}\in\mathcal{O}} \ , \\ \mathbf{B}(p) &:= \begin{pmatrix} p^2 & p(1-p) & p(1-p) & (1-p)^2 \\ p & 0 & 0 & 1-p \end{pmatrix} \ , \end{aligned} \tag{17}$$

are the conditional probabilities of the observed haplotypes given the true ones,

$$\begin{aligned} e_{\mathbf{i},\mathbf{j}}(\varepsilon) &= P\left(O_k = \mathbf{j} | H_k = \mathbf{i}\right) = P\left(O_k^{\mathcal{A}} = j^{\mathcal{A}} | H_k^{\mathcal{A}} = i^{\mathcal{A}}\right) P\left(O_k^{\mathcal{B}} = j^{\mathcal{B}} | H_k^{\mathcal{B}} = i^{\mathcal{B}}\right) \\ &= h_{j^{\mathcal{A}},i^{\mathcal{A}}} h_{j^{\mathcal{B}},i^{\mathcal{B}}}\,, \quad \mathbf{i},\mathbf{j} \in \mathcal{O}\,, \qquad (18a) \\ h_{t,s} &:= \varepsilon + (1-2\varepsilon)\delta_{t,s}\,, \qquad t,s = 0,1\,. \qquad (18b) \end{aligned}$$

is a genotype error model that assumes errors occur in the alleles independently of each other, $\delta_{t,s}$ is Kronecker's delta, and $\varepsilon = 0.01$ is the expected genotype error rate.

The initial state distribution vector is the unconditional probability of IBD, $\mathbf{f}$. Note that the state transition from SNV k to $k+1$ is a function of the genetic distance between the markers, while the emission at SNV k depends on the allele frequencies thereof. Notwithstanding, the same forward-back algorithm [12] can still be applied to calculate

$$\begin{aligned} \alpha_k(i) &:= P\left(O_1, \ldots, O_k, S_k = i\right)\,, \qquad (19a) \\ \beta_k(i) &:= P\left(O_{k+1}, \ldots, O_n, S_k = i\right)\,, \qquad (19b) \\ \gamma_k(i) &:= P\left(S_k = i | \mathbf{O}\right) = \frac{\alpha_k(i)\beta_k(i)}{\sum_{i'=0}^{1} \alpha_k(i')\beta_k(i')}\,. \qquad (19c) \end{aligned}$$

The values $\{\gamma_k(1)\}_{k=1}^n$ are the desired IBD posterior at the markers. In practice, deciding on IBD via thresholding the posterior frequently breaks a longer IBD segments into shorter streteches, if the posterior fluctuates about the threshold. Thus, we instead use the Viterbi path (computed using the Viterbi algorithm [12]) to detect IBD segments. The path seems to be more robust to changes in λ and does not require a threshold. However, within the IBD segment, we quantify the local certainty of IBD at SNV k by $\gamma_k(1)$, which is subsequently used in deciding which segment is used for phasing and imputation.

.2 Genotype vs. Genotype

We define the analogous HMM to the HH case, which is similar to IBDLD without LD modeling [7]. Here O_k^l, H_k^l have three possible values, $0, 1$ or 2, and S_k is the condensed identity state.

Uricchio et al. [15] assumed a constant state S over the entire frame to simplify the posterior estimation. However, it is important to allow local state transitions, because S_k may change even within a true (IBD ≥ 1)-segment in the presence of recombinations, as illustrated by Figure S14.

While this cannot occur in the HH case, the joint evidence provided by all SNVs is always more accurate than single SNVs and will likely produce better IBD estimation (at an extra cost, of course).

The initial state distribution is $\Delta := (\Delta_1, \ldots, \Delta_9)$. The transition matrix is again defined by the infinitesimal transition rate stationary distribution model [7]

$$\mathbf{A}(x) = \Delta \mathbf{1}^T + e^{-\lambda x}\left(\mathbf{I}_9 - \Delta \mathbf{1}^T\right)\,, \qquad (20)$$

where $\mathbf{1} = \underbrace{(1, \ldots, 1)}_{9}^T$ and $\mathbf{I}_n$ is the $n \times n$ identity matrix. The parameter $\lambda \geq 0$ is specific to the subjects $\mathcal{A}, \mathcal{B}$. We use the value 0.4λ, where λ is the quantity computed for the Hutterites subjects by Abney et al. as the best-fit in Monte-Carlo gene dropping simulations [7]. This seems to reduce undesired state transitions for short SNV stretches within true IBD segments between close relatives.

The emission probabilities are given by (16), where now $\{b_{i,\mathbf{j}}\}_{i,\mathbf{j}}$ are the values in Table S2; the error model is still (18b), but here j^l, i^l range over $0, 1, 2$ for each $l = \mathcal{A}, \mathcal{B}$, and $\{h_{s,t}\}_{s,t}$ are given by Table S4 [7, Table S2].

The Viterbi path is used as the indicator of IBD (i.e., if the state is 1,3,5,7, or 8, we infer that the SNV is IBD ≥ 1, otherwise IBD $= 0$). Once an IBD segment is determined, we do quantify the local IBD certainty at each SNV by

$$P(\text{IBD} \geq 1 \text{ at SNV } k | \mathbf{T} = \mathbf{t}) = \sum_{i \in \{1,3,5,7,8\}} P\left(S_k = i | \mathbf{T} = \mathbf{t}\right) = \sum_{i \in \{1,3,5,7,8\}} \gamma_k(i)\,, \qquad (21)$$

which is computed by the forward-backward algorithm (19). Figure S15 illustrates both indicators.

IBD Cliques at a SNV

After phasing, we apply the HMM of Sec. IBD Estimation in a Segment, Haplotype vs. Haplotype to each pair of haplotypes among the 1415 Hutterites and identify pairwise IBD segments. We then organize segments in an *IBD segment index* data structure, which consists of a set of IBD cliques at each SNV. In this section we describe a method for defining IBD cliques from pairwise IBD segment at a certain SNV k.

We build a weighted, undirected pairwise IBD graph $G = (\mathcal{N}, \mathcal{E}, w)$, where $\mathcal{N}$ is the set of $n = 2830$ haplotypes and $m := |\mathcal{E}|$ edges. An edge $(u, v) \in \mathcal{E}$ exists if and only if haplotypes u and v are IBD at the SNV. The weight function $w : \mathcal{E} \to [0, 1]$ is the posterior HMM IBD probability $\gamma_k(1)$ (19c).

Because IBD is a transitive relation, G must be a union of disjoint cliques (i.e., fully connected sub-graphs), one for each ancestral haplotype present in the population. In practice, G is a perturbation of a clique union due to very low HMM certainty near segment ends and genotyping errors, and we would like to recover a "reasonable" set of cliques from it. Cluster editing methods (see for instance [13], but the literature is quite rich) solve the problem of finding the minimum number of edges (or total edge weight) that need to be added or removed to transform G to a clique union. This is an NP-hard problem, and practical heuristic-based algorithms run in superlinear time ($O(m^2)$ or slower). We choose a different heuristic inspired by the graph algebraic multigrid literature [14, 11, 3] that resulted in good imputation cross-validation accuracy and requires only $O(m)$ time and storage.

The Laplacian matrix $\mathbf{A}_{n\times n}$ is defined by

$$\mathbf{A} = (a_{uv})_{u,v} \ , \quad a_{uv} := \begin{cases} \sum_{v'\in\mathcal{E}_u} w_{uv'} \ , & u = v \ , \\ -w_{uv} \ , & v \in \mathcal{E}_u := \{v' : (u,v') \in \mathcal{E}\} \ , \\ 0 \ , & \text{otherwise.} \end{cases} \tag{22}$$

Note that $\mathbf{A} = \mathbf{D} - \mathbf{W}$, where $\mathbf{W}$ is the adjacency matrix and $\mathbf{D} := \mathrm{diag}(\mathbf{W})$. The associated *quadratic energy* is

$$E(\mathbf{x}) := \mathbf{x}^T \mathbf{A} \mathbf{x} = \sum_{(u,v)\in\mathcal{E}} w_{uv} \left(x_u - x_v\right)^2 \ , \qquad \mathbf{x} \in \mathbb{R}^{\mathcal{N}} \ , \tag{23}$$

Clique are identified in three steps:

1. Generate K smooth (low-energy) Test Vectors (TVs) $\mathbf{x}^{(1)}, \ldots, \mathbf{x}^{(K)}$ [11, §3.3.1]. Each TV is the result of applying ν damped-Jacobi relaxation sweeps to the sparse linear system $\mathbf{Ax} = \mathbf{0}$, starting from random$[-1,1]$, i.e.,

$$\mathbf{x}^{(k)} = \left((1-\omega)\mathbf{I} + \omega \mathbf{D}^{-1}\mathbf{W}\right)^{\nu} y \ , \quad y_u = \mathrm{random}[-1,1] \ , u \in \mathcal{N} \ , \quad k = 1, \ldots, K \ . \tag{24}$$

   We use $K = 5, \nu = 5$ and $\omega = 0.7$.

2. Calculate new edge weights called *affinities* [11, §3.3.2] that measure node similarity. The affinity c_{uv} between u and v is defined as the goodness of fitting the linear model $x_v \approx p\, x_u$ to TV values:

$$c_{uv} := 1 - \frac{\left|(X_u, X_v)\right|^2}{(X_u, X_u)\,(X_v, X_v)} \ , \quad (X, Y) := \sum_{k=1}^{K} x^{(k)} y^{(k)} \ , \quad X_u := \left(x_u^{(1)}, \ldots, x_u^{(K)}\right) \ . \tag{25}$$

3. Remove edges with $\omega_{uv} < 0.85$ or $c_{uv} < 0.9$ from G.

4. Define the cliques to be the connected components of the modified graph.

The affinity c_{uv} is a crude approximation of the *diffusion distance* at a short time ν [5]. A large affinity indicates that the nodes are strongly connected not just through the direct edge between them, but also through many short paths: their neighborhoods in the graph are similar. The essential practical point is that this crude and inexpensive "algebraic distance" is all that is needed to generate a good coarsening of the graph. In this case, the graph contains only two scales: the haplotype scale and the clique scale.

This approach needs to be further investigated and compared with other methods. Damped Jacobi was chosen as a smoother because of its parallelizability, but Gauss-Seidel [11, §2] is a better smoother. The choice of the various parameters K, ν, ω followed [11], we don't include a simple receipe for choosing the optimal edge weight thresholds

in step 3. We chose them based on specific SNV data in which we manually identified the cliques from the pedigree structure, and our ultimate performance measure was the imputation cross-validation accuracy. In general, the threshold should depend on the level of smoothness of the test vectors (i.e., on K and ν) but should apply to the entire class of IBD graphs that have a two-scale structure.

Tables

Algorithm	Step	Complexity	CPU Hours
Pedigree-based Imputation (PRIMAL)	Phase	$O(f^2 s)$	1,400
	Find IBD segments	$O(n^2 s)$	$48,000$
	Index segments into cliques	$O(n^2 s)$	$24,000$
	Assign parental origin	$O(ns)$	$1,400$
	Impute	$O(ns)$	140
	Total	$O(n^2 s)$	$74,940$
LD-Based Imputation	IMPUTE2	$O(n^s)$	60
PRIMAL+LD		$O(n^2 s)$	$75,000$

Table S1: PRIMAL algorithm worst-case complexity vs. the number of genotyped subjects ($n = 1,415$), number of framework markers ($s = 271,486$), average family size in pedigree ($f = 4.1$) and actual run times for the Hutterite data set on the Beagle supercomputer.

		$\mathbf{j}$	$(0,0)$	$(2,2)$	$(0,2)$	$(2,0)$	$(0,1)$	$(2,1)$	$(1,0)$	$(1,2)$	$(1,1)$
		$IBS \rightarrow$	2		0		1		1		2
i	State	IBD $\downarrow$	$b_{i,\mathbf{j}}$								
1	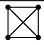	2	p	q	0	0	0	0	0	0	0
2	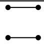	0	p^2	q^2	pq	pq	0	0	0	0	0
3	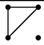	1	p^2	q^2	0	0	pq	pq	0	0	0
4	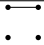	0	p^3	q^3	pq^2	p^2q	$2p^2q$	$2pq^2$	0	0	0
5	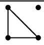	1	p^2	q^2	0	0	0	0	pq	pq	0
6	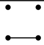	0	p^3	q^3	p^2q	pq^2	0	0	$2p^2q$	$2pq^2$	0
7	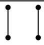	2	p^2	q^2	0	0	0	0	0	0	$2pq$
8	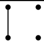	1	p^3	q^3	0	0	p^2q	pq^2	p^2q	pq^2	pq
9	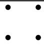	0	p^4	q^4	p^2q^2	p^2q^2	$2p^3q$	$2pq^3$	$2p^3q$	$2pq^3$	$4p^2q^2$

Table S2: The conditional probabilities $b_{i,\mathbf{j}} := P(G = \mathbf{j}|S = i)$.

j	$\xi_t(p)$	$\eta_t(p)$
$(0,0)$	$\Delta_1 + p(\Delta_3 + \Delta_5 + \Delta_7) + p^2\Delta_8$	$p\left(\Delta_2 + p(\Delta_4 + \Delta_6) + p^2\Delta_9\right)$
$(0,1)$	$\Delta_3 + p\Delta_8$	$2p\left(\Delta_4 + p\Delta_9\right)$
$(1,0)$	$\Delta_5 + p\Delta_8$	$2p\left(\Delta_6 + p\Delta_9\right)$
$(1,1)$	$\Delta_7 + 2\Delta_8$	$2p(1-p)\Delta_9$

Table S3: The terms of Eq. (10) for the case $t > 0, t \neq 2$.

	$t=0$	$t=1$	$t=2$
$s=0$	$(1-\varepsilon)^2$	$2\varepsilon(1-\varepsilon)$	ε^2
$s=1$	$\varepsilon(1-\varepsilon)$	$\varepsilon^2+(1-\varepsilon)^2$	$\varepsilon(1-\varepsilon)$
$s=2$	ε^2	$2\varepsilon(1-\varepsilon)$	$(1-\varepsilon)^2$

Table S4: The conditional probabilities $h_{s,t} := P(O=t|G=s)$, where O is the observed genotype and G is the true genotype.

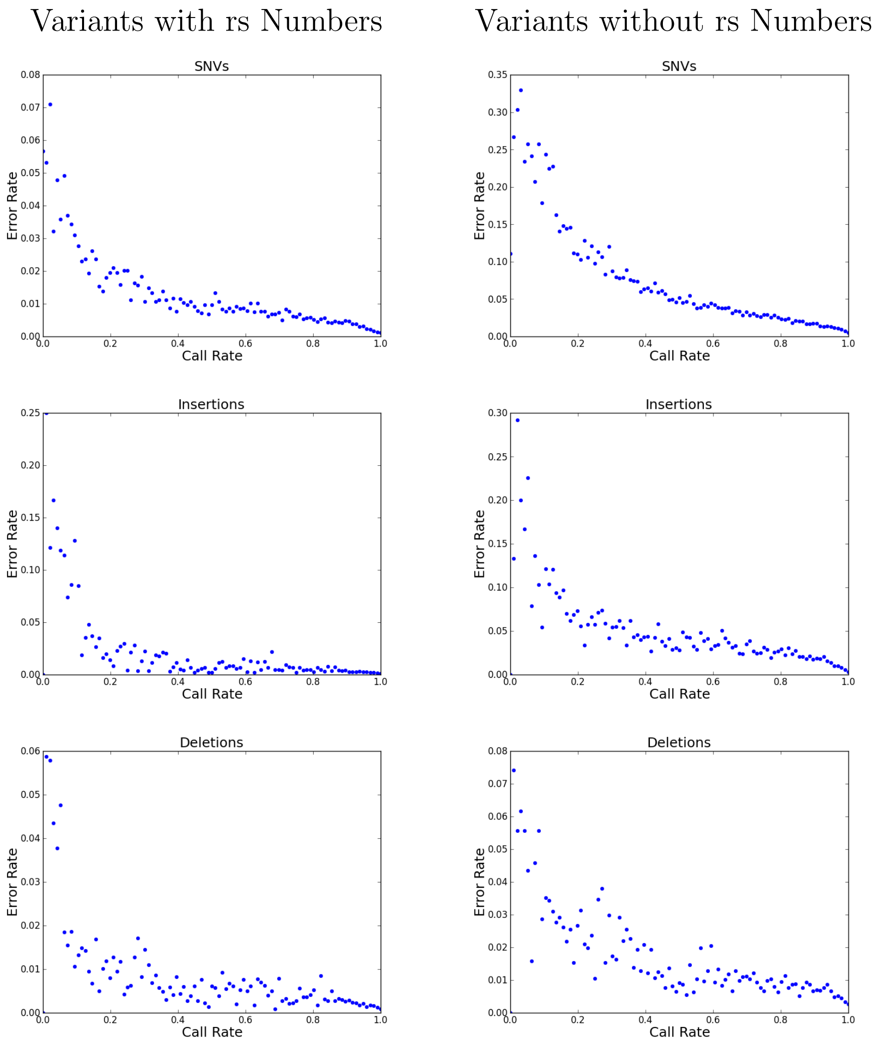

Figure S1: Generalized Mendelian error rate (the total number of discordances divided by the total number of IBD2 segments) vs. call rate (fraction of called genotypes among the 98 whole genome sequences) for non-singleton variants, shown by variant type (SNVs, insertions, deletions) and novelty (with or without rs numbers).

Figures

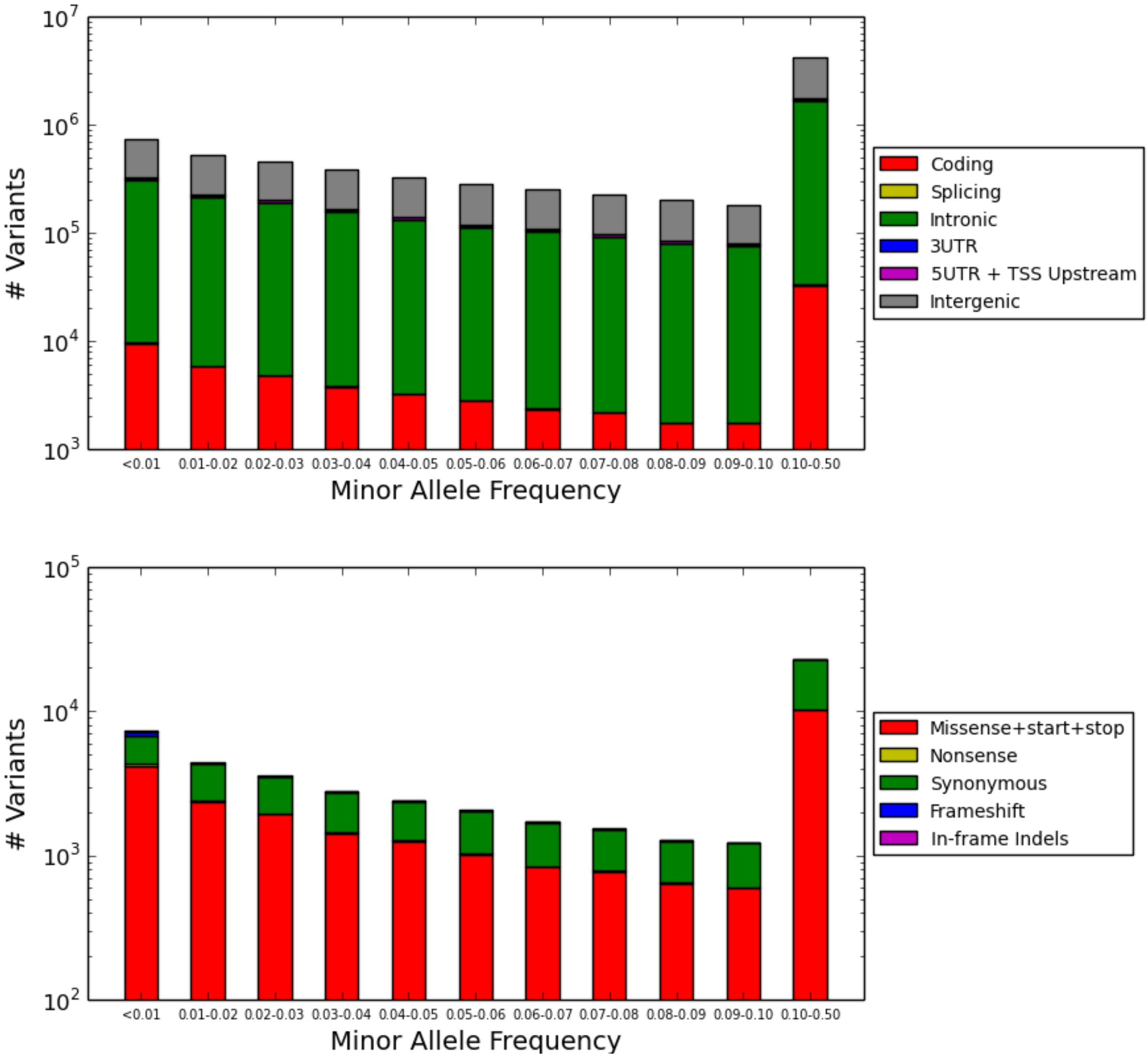

Figure S2: Distribution of variants present in 98 Hutterite genome sequences by frequency and functional class. Top: all variants. Bottom: variants in coding regions.

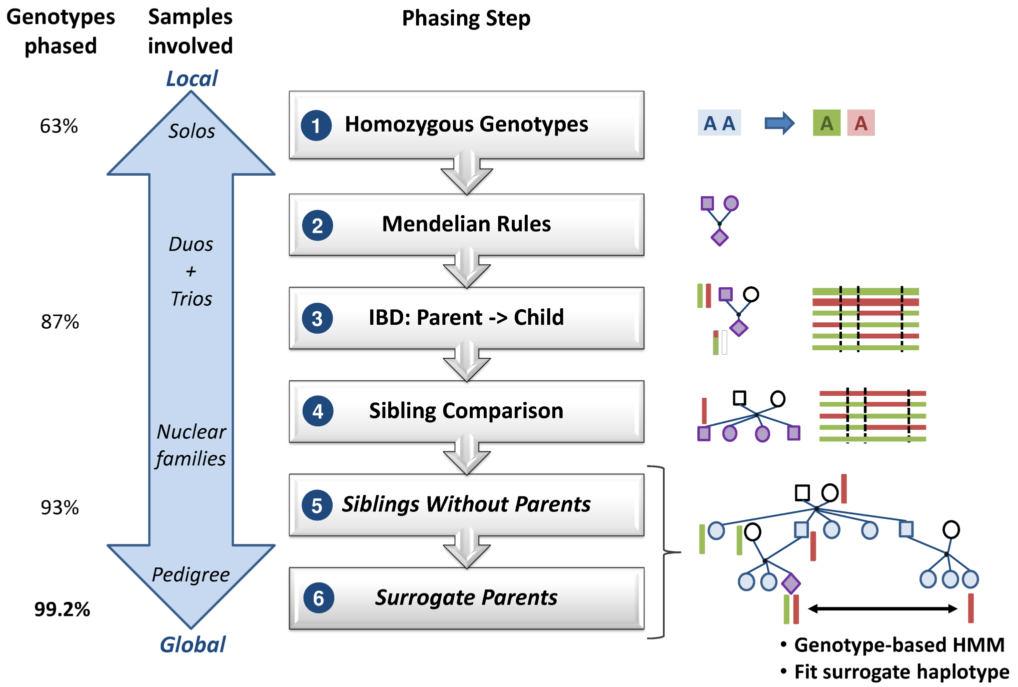

Figure S3: Phasing algorithm. First, we phase homozygous SNVs (1) and SNVs that are homozygous in an individual's parent (2). In trios with phased parents, we phase more SNVs using parent-child IBD segments (3). In large families with genotyped parents, we compare offspring haplotypes to obtain child-child segments and phase any unphased sibling. For the remaining subjects, we use a Hidden Markov Model (HMM) to identify IBD segments between the individual's genotype and a surrogate parent's haplotype (5-6).

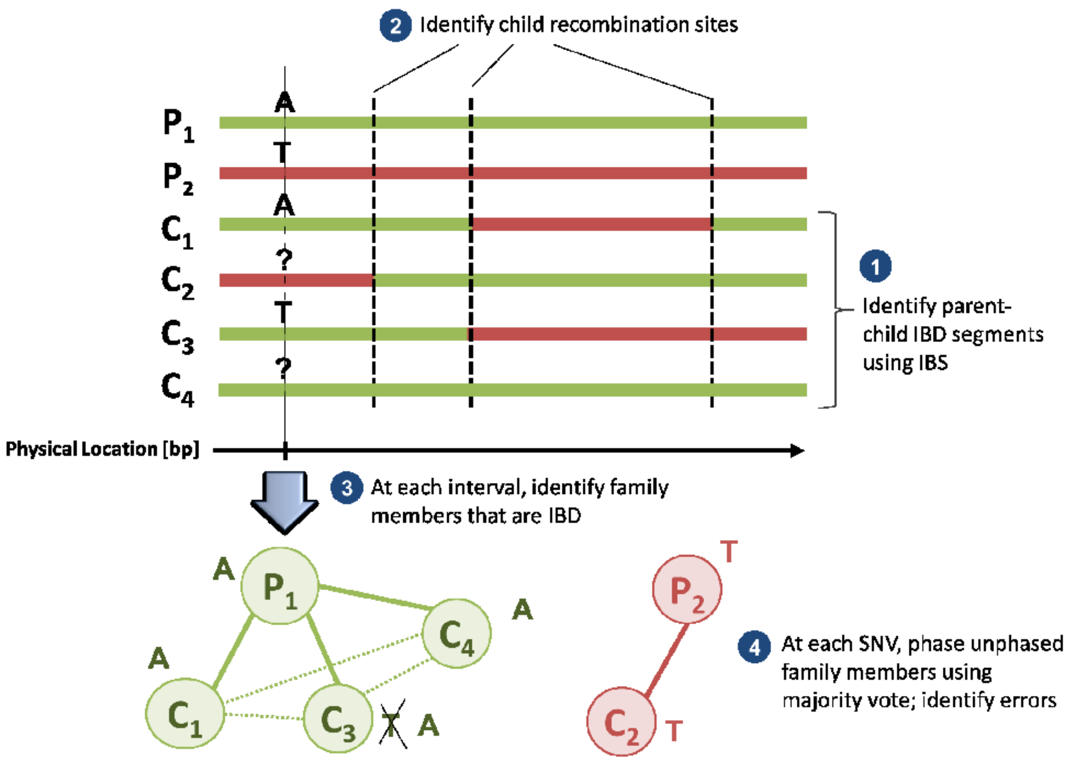

Figure S4: Phasing Step 3. Given a father P who is phased at most heterozygous SNVs, call his partially-known haplotypes P_1 and P_2 and $C_1, \ldots, C_4$ be the partially known paternal haplotypes of his four children. We first determine IBD segments between P and each child using long IBS stretches (1), identify child recombination sites (vertical dashed blue lines) (2), and determine which set of children is IBD with P_1 and P_2 in each interval between recombination sites (3). Finally, at each SNV, we apply a majority vote (4) within each IBD haplotype clique to phase unphased members of the clique (C_2 and C_4 in this case) and correct errors in others (such as in C_3).

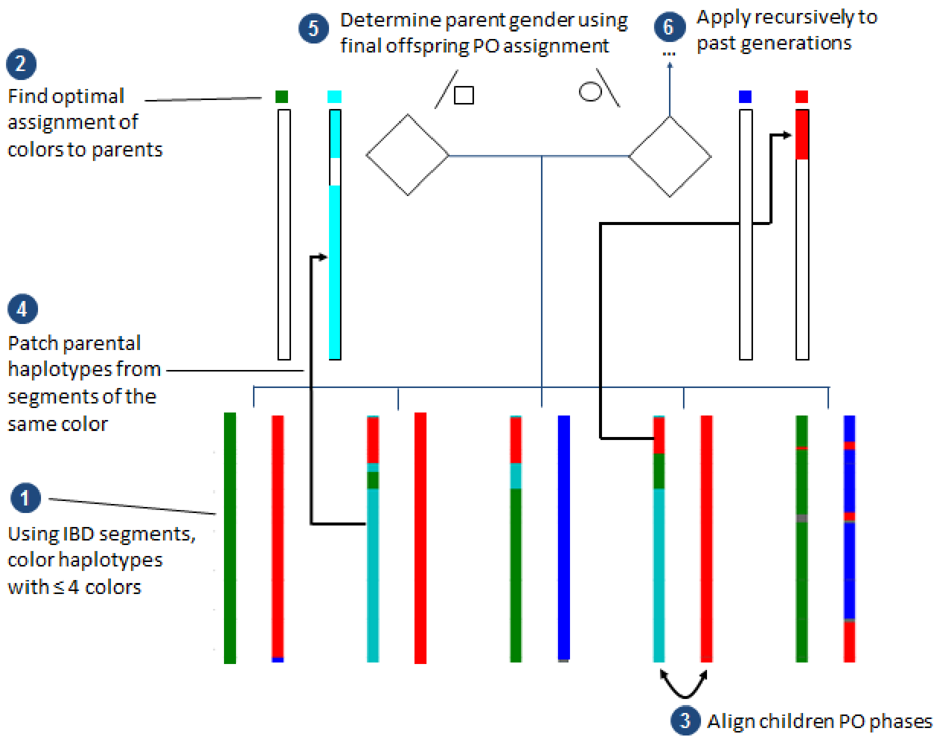

Figure S5: Untyped ancestor imputation. (1) Using IBD segments discovered among quasi-founder siblings, we color their haplotypes with at most four colors representing the four parental haplotypes, so that the number of recombinations is minimized. (2) Of the three possible assignments of color pairs to parents, we pick the one that yields the clearest separation of offspring haplotypes into paternal and maternal. This induces the consistent ordering of offspring haplotypes ("PO phase alignment") as paternal first, maternal second, for example (3). (4) Segments of the same color are patched to reconstruct each parental haplotype. (5) Parent gender is determined by the PO clique method (Figure 9). Finally, the same methodology can be applied iteratively to impute members of earlier generations (6).

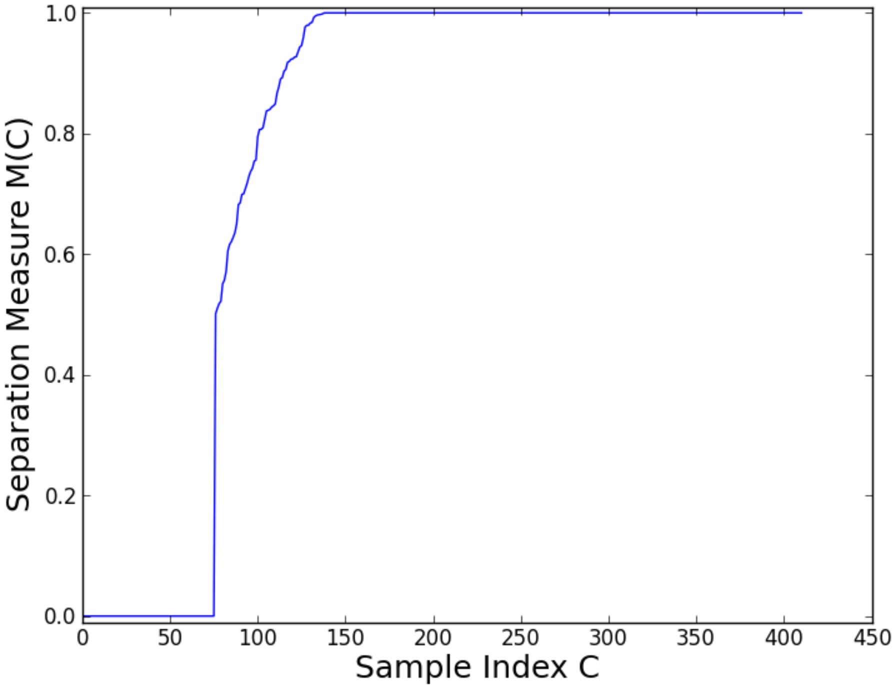

Figure S6: Parental origin separation measure M(C) for chromosome 22 in 411 Hutterite quasi-founders, sorted ascending by M-value. Parental origin was assigned to the haplotypes of 313 quasi-founders with $M(C) > 0.75$ as well as to the haplotypes of all $1,004$ non-quasi founders (using Mendelian rules applied during phasing). In total, we could assign parental origin to 93% ($1,317$ out of $1,415$) subjects.

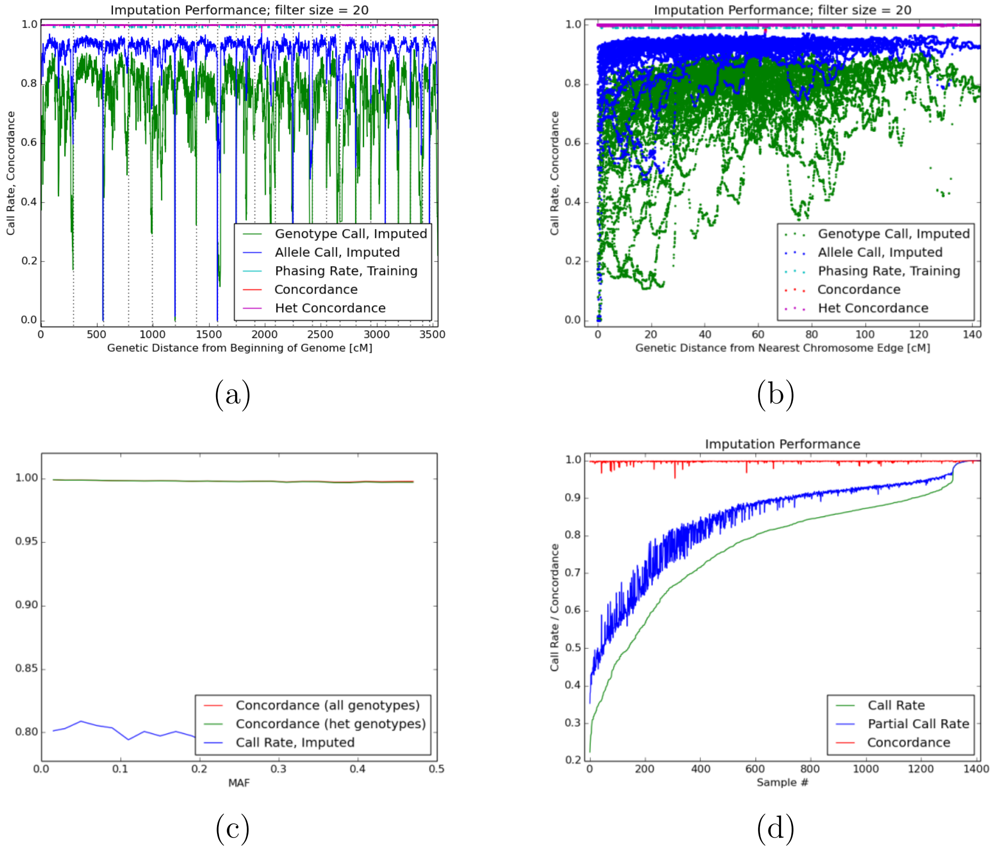

Figure S7: PRIMAL cross validation on a sample of 53,861 of framework SNVs with MAF $\geq 1\%$. (a) Genotype call rates and concordance along the genome. (b) Genotype call rates and concordance along the genome versus genetic distance from the nearest chromosome edge. (c) Genotype call rates and concordance versus minor allele frequency. (d) Genotype call rates and concordance, sorted by ascending genotype call rate.

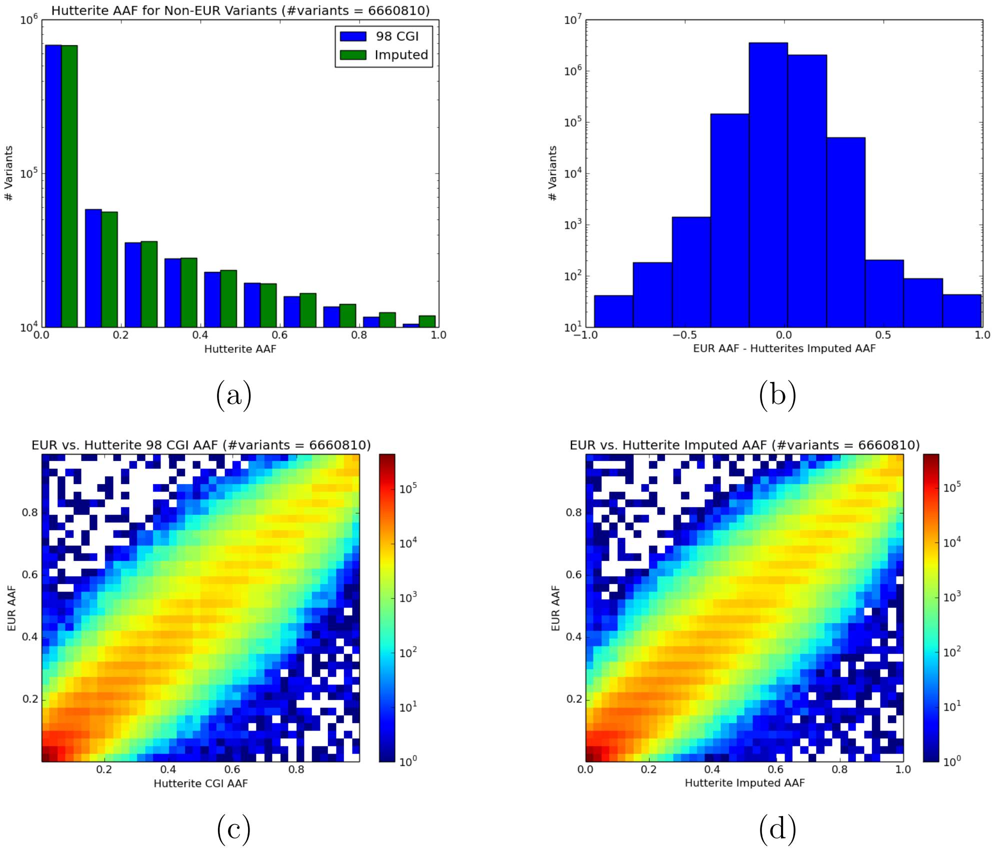

Figure S8: (a) Alternative Allele Frequency (AAF) distribution in 98 sequenced Hutterites (blue) and in the 1,415 sequenced and imputed Hutterites (green), for variants that are ultra rare in European populations. (b) Frequency of the difference between the EUR AAF and Hutterites imputed AAF. (c) AAF in the 98 sequenced Hutterites vs. the 1000Genomes EUR subjects. (d) AAF in the 1,415 sequenced and imputed Hutterites vs. 1000Genomes EUR subjects.

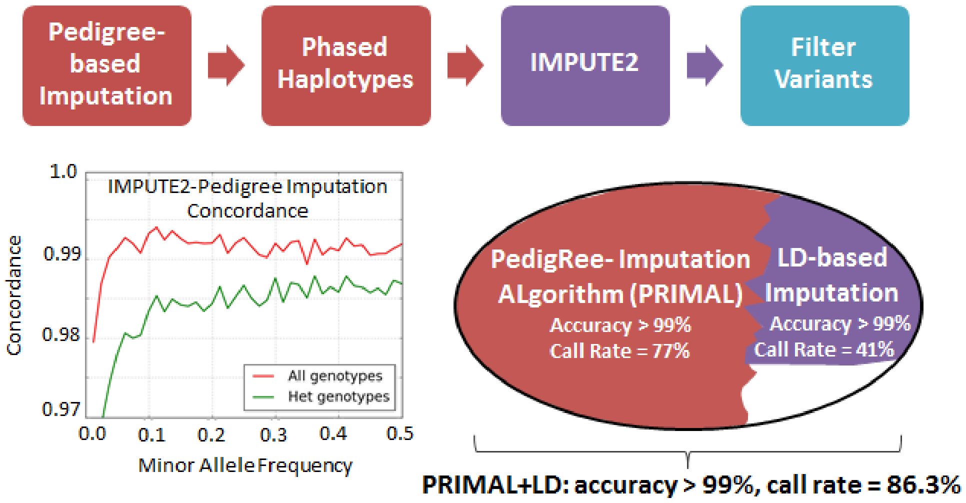

Figure S9: IMPUTE2-Pedigree-based concordance. After filtering variants by the criterion het concordance $\geq 98\%$ and MAF $\geq 1.5\%$, the LD-based imputation call rate is 56% out of the remaining 23% not imputed by PRIMAL, providing a combined call rate of 89% at the same accuracy of the pedigree-based method ($\geq 99\%$).

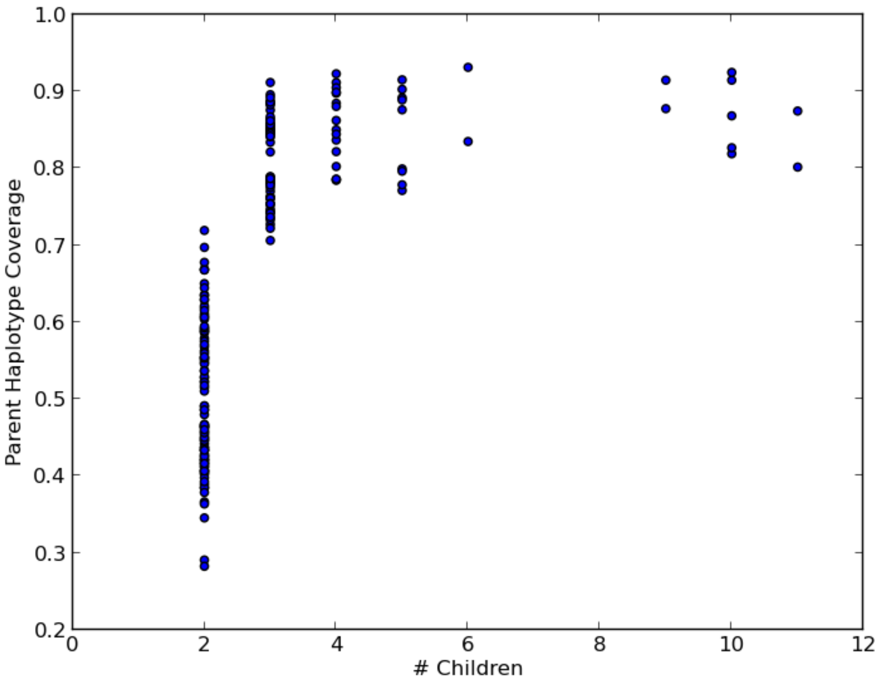

Figure S10: Fraction of haplotype coverage of quasi-founder parents vs. number of offspring.

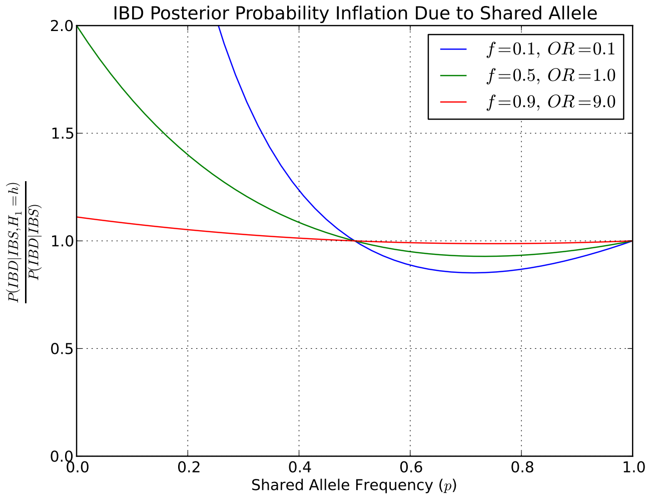

Figure S11: The ratio of Eq. (6) to (7) vs. p and f.

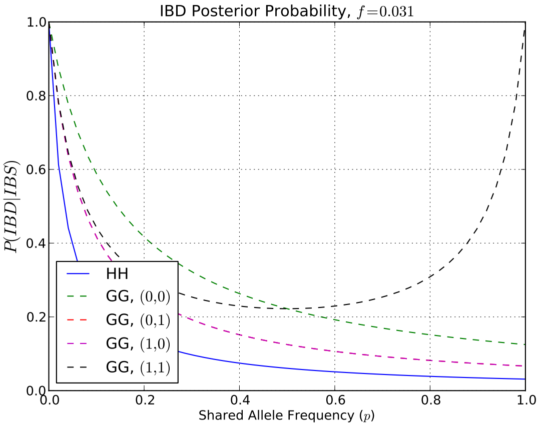

(a) Outbred, $f = 1/32$

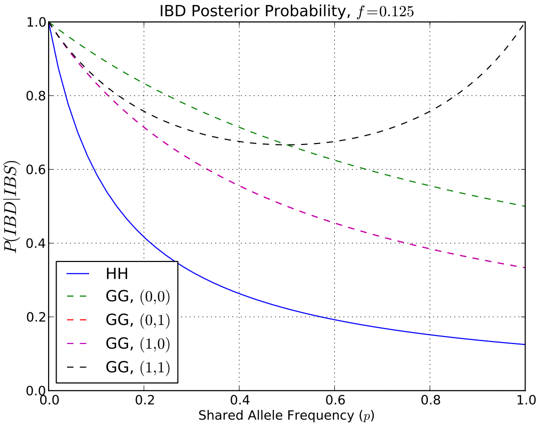

(b) Outbred, $f = 1/8$

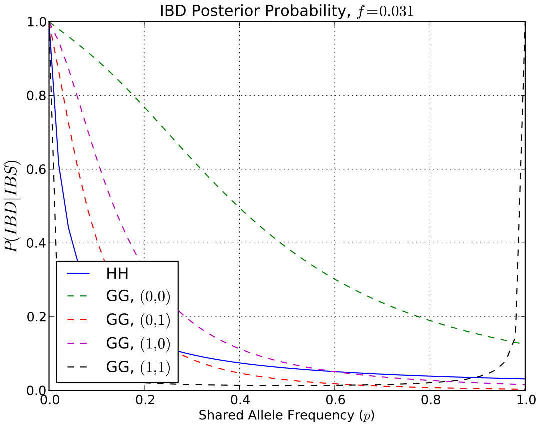

(c) Inbred, $f = 1/32$

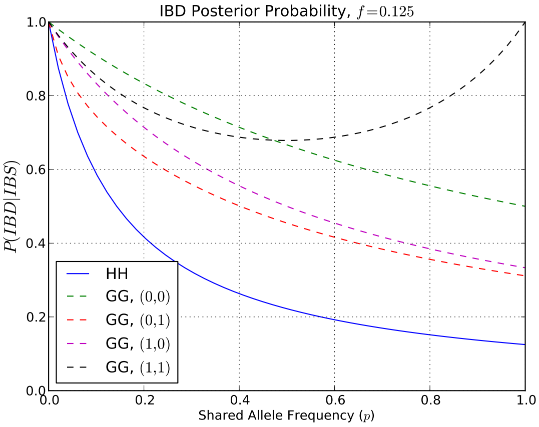

(d) Inbred, $f = 1/8$

Figure S12: IBD posterior probability for haplotype-haplotype data (denoted HH; Eq. (6)), and genotype-genotype data (GG – dashed lines; Eq. (10)) vs. p and f.

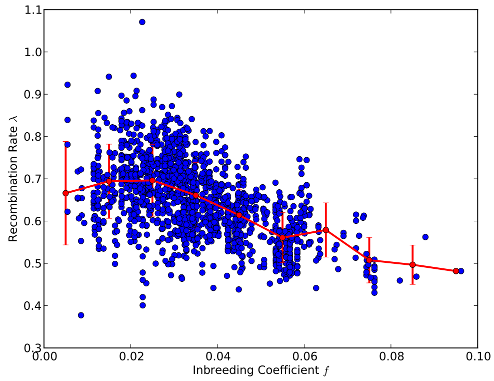

(a)

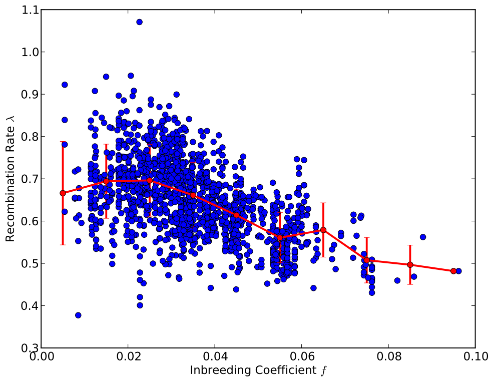

(b)

Figure S13: (a) Genotype transition rate λ vs. the in-breeding coefficient f in the Hutterites (blue scatter) and mean and standard deviation over bins of similar f-values (red error bars). (b) The standard deviation of λ among siblings vs. their mean λ, for all genotyped Hutterites families.

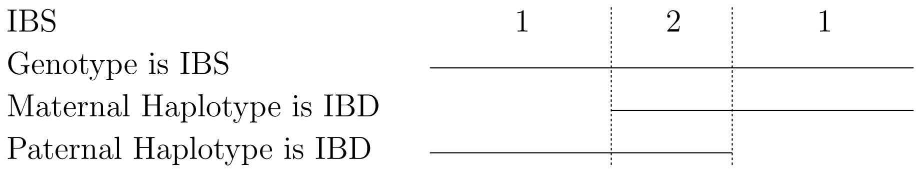

Figure S14: An IBS segment that is partially IBD in the paternal haplotype and partially IBD in the maternal haplotype, causing a transitions in the identity state. A line indicates that the corresponding event occurs in that SNV range.

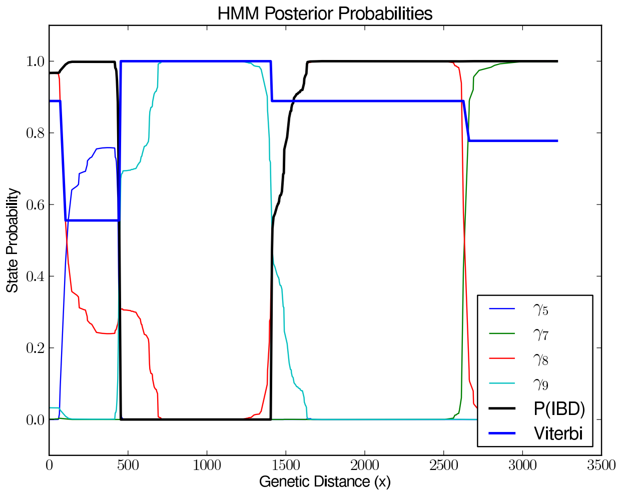

Figure S15: HMM results for a pair of Hutterite subjects. Only states i with significant $\gamma.(i)$ values are included. The Viterbi path is recoded from states $1, 2, \ldots, 9$ to the values $1/9, 2/9, \ldots, 1$, so that it is more clearly overlayed over the same range.

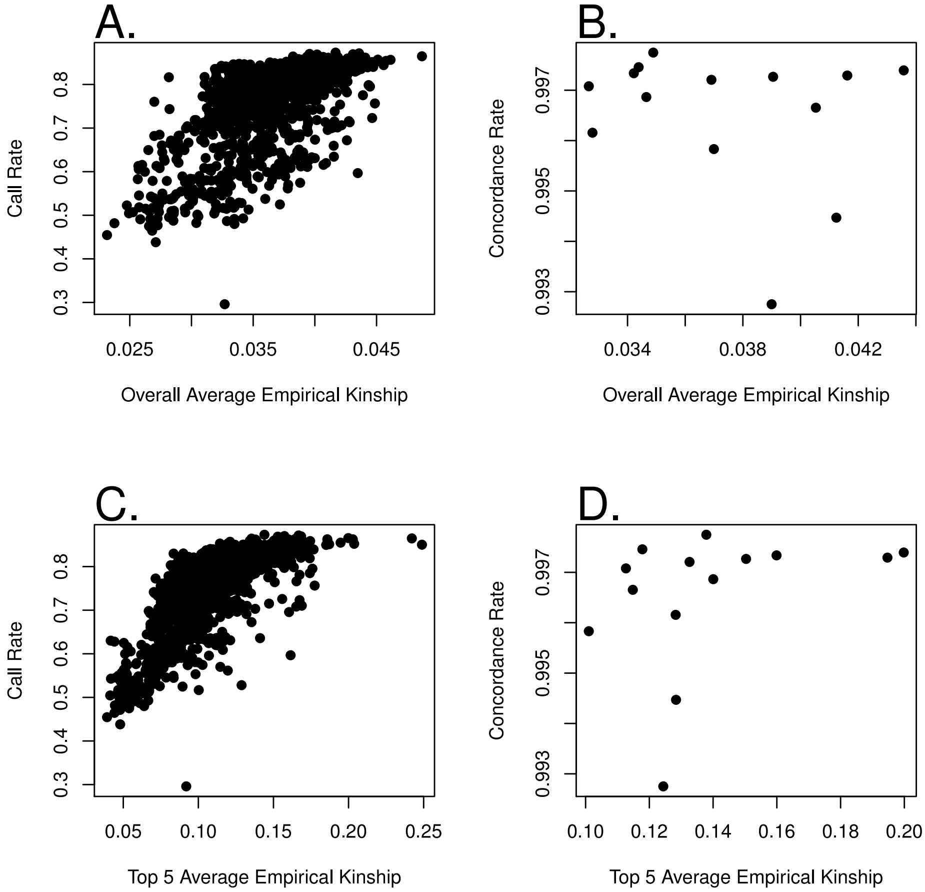

Figure S16: Scatter plots of relationship between concordance or call rates and empirical kinship metrics are shown. Panels A and C show the call rates for the 1317 imputed Hutterites and two measures of relatedness with the 98 sequenced individuals: average empirical (from observed IBD sharing) kinship (in panel A) and average of the closest five (with respect to kinship) sequenced subjects (in panel C). Panels B and D show the concordance rates for the 14 subjects who were validated using sequencing with an independent platform. As expected, Hutterites who are more related to the sequenced individuals tend to have higher call rates and better imputation accuracy.

References

[1] M. Abney. Identity-by-descent estimation and mapping of qualitative traits in large, complex pedigrees. *Genetics*, 179(3):1577–1590, 2008. 10.1534/genetics.108.089912.

[2] M. Abney, C. Ober, and M.S. McPeek. Quantitative trait homozygosity and association mapping and empirical genome-wide significance in large complex pedigrees: Fasting serum insulin levels in the Hutterites. *Amer. J. Hum. Gen.*, 70:920–934, 2002.

[3] A. Brandt, J. Brannick, K. Kahl, and I. Livshits. An algebraic distances measure of AMG strength of connection. ArXiV e-prints, `http://arxiv.org/abs/1106.5990v1`, 2011.

[4] S. R. Browning. Estimation of pairwise identity by descent from dense genetic marker data in a population sample of haplotypes. *Genetics*, 178(4), 2008.

[5] R.R. Coifman and S. Lafon. Diffusion maps. *Applied Comput. Harmon. Anal.*, 21:5–30, 2006.

[6] G. Coop, X. Wen, C. Ober, J. K. Pritchard, and M. Przeworski. High-resolution mapping of crossovers reveals extensive variation in fine-scale recombination patterns among humans. *Science*, 319, 2008.

[7] L. Han and M. Abney. Identity by descent estimation with dense genome-wide genotype data. *Genetic Epidemiology*, 35(6):557–567, 2011.

[8] R.V. Hogg and E. Tanis. *Probability and Statistical Inference.* Prentice Hall, 2009.

[9] A. Kong, G. Masson, M. L. Frigge, A. Gylfason, P. Zusmanovich, G. Thorleifsson, P. I. Olason, A. Ingason, S. Steinberg, T. Rafnar, P. Sulem, M. Mouy, F. Jonsson, U. Thorsteinsdottir, D. F. Gudbjartsson, H. Stefansson, and K. Stefansson. Detection of sharing by descent, long-range phasing and haplotype imputation. *Nature Genetics*, 40, 2008.

[10] K. Lang. *Mathematical and Statistical Methods for Genetic Analysis.* Springer, 2002.

[11] O.E. Livne and A. Brandt. Lean algebraic multigrid (LAMG): Fast graph laplacian linear solver. *SIAM J. Sci. Comput.*, 34(4):B499–B522, 2011.

[12] L. R. Rabiner. A tutorial on Hidden Markov Models and selected applications in speech recognition. In *Proceedings of the IEEE*, pages 257–286, 1989.

[13] S. Rahmann, T. Wittkop, J. Baumbach, M. Martin, Truss A., and S. Bocker. Exact and heuristic algorithms for weighted cluster editing. *Comput. Syst. Bioinformatics Conf.*, 6, 2007.

[14] D. Ron, I. Safro, and A. Brandt. Relaxation-based coarsening and multiscale graph organization. *Multiscale Model. Sim.*, 9(1):407–423, 2011.

[15] L. H. Uricchio, J. X. Chong, K. D. Ross, C. Ober, and D. L. Nicolae. Accurate imputation of rare and common variants in a founder population from a small number of sequenced individuals. *Genetic Epidemiology*, 36(4):312–319, 2012.
